# Supplementary material for: Dual-quartet phosphorescent emission in the open-shell M1Ag13 (M = Pt, Pd) nanoclusters
Source: Nat Commun. 2024 Jul 16;15:5962. doi: 10.1038/s41467-024-50289-x (PMC11252300; doi:10.1038/s41467-024-50289-x)
Supplement: Supplementary file 1 — Supplementary Information [file 41467_2024_50289_MOESM1_ESM.pdf]

Supplementary Information

Dual-quartet phosphorescent emission in the open-shell M_1Ag_{13} (M = Pt, Pd) nanoclusters

Cao Fang[1,2,5], Chang Xu[1,5], Wei Zhang[3], Meng Zhou[3], Dong Tan[1,2], Lixia Qian[1], Daqiao Hu[1,2]*, Shan Jin[2,4]* & Manzhou Zhu[1,2]*

[1] Department of Chemistry and Centre for Atomic Engineering of Advanced Materials, Anhui University, Hefei, Anhui 230601, China. [2] Key Laboratory of Structure and Functional Regulation of Hybrid Materials of Ministry of Education, Anhui University, Hefei, Anhui 230601, China. [3] Hefei National Laboratory for Physical Sciences at the Microscale, University of Science and Technology of China, Hefei, Anhui 230026, China. [4] Institutes of Physical Science and Information Technology, Anhui University, Hefei, Anhui 230601, China. [5] These authors contributed equally: Cao Fang, Chang Xu. *E-mail: hudaqiao@ahu.edu.cn; jinshan@ahu.edu.cn; zmz@ahu.edu.cn

This Supplementary Information includes:

Supplementary Figures 1-40

Supplementary Tables 1-14

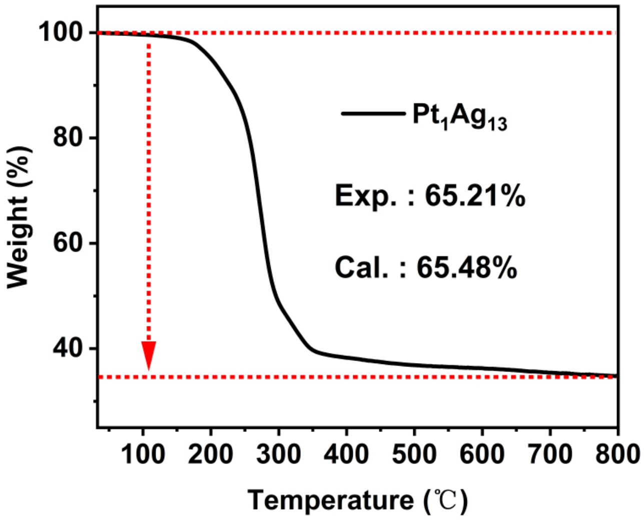

Supplementary Figure 1. Thermogravimetric analyses (TGA) curves of Pt_1Ag_{13} in N_2 atmosphere.

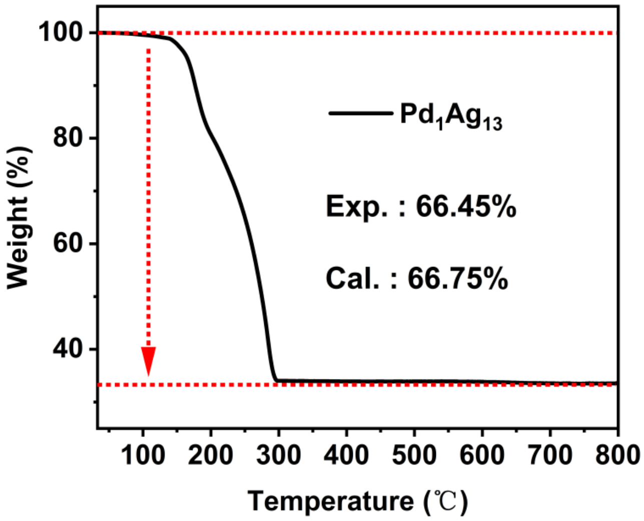

Supplementary Figure 2. Thermogravimetric analyses (TGA) curves of Pd_1Ag_{13} in N_2 atmosphere.

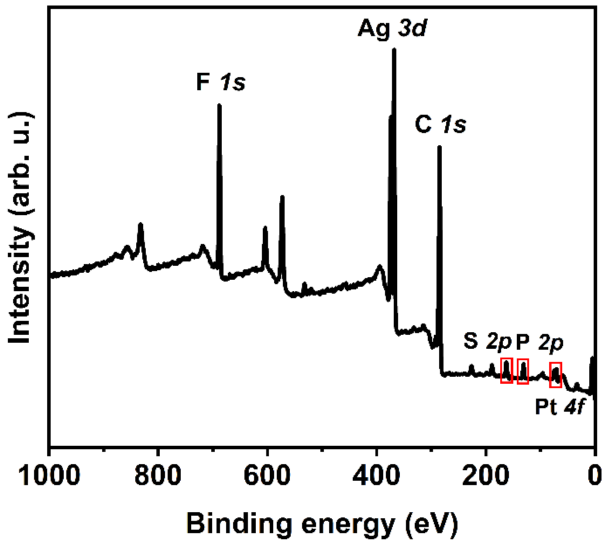

Supplementary Figure 3. The XPS survey of Pt_1Ag_{13}.

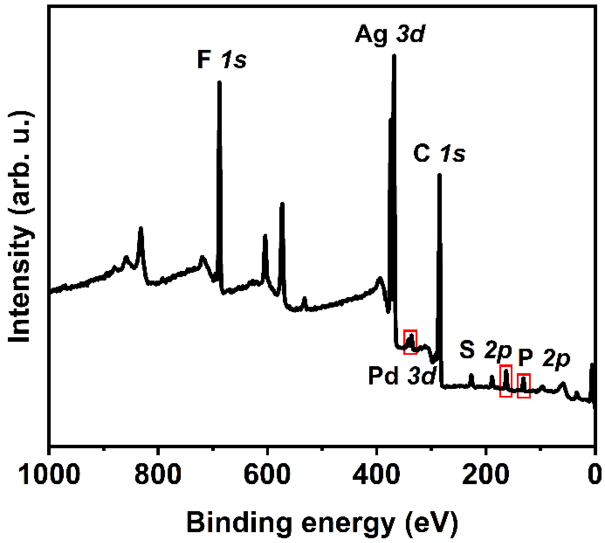

Supplementary Figure 4. The XPS survey of Pd_1Ag_{13}.

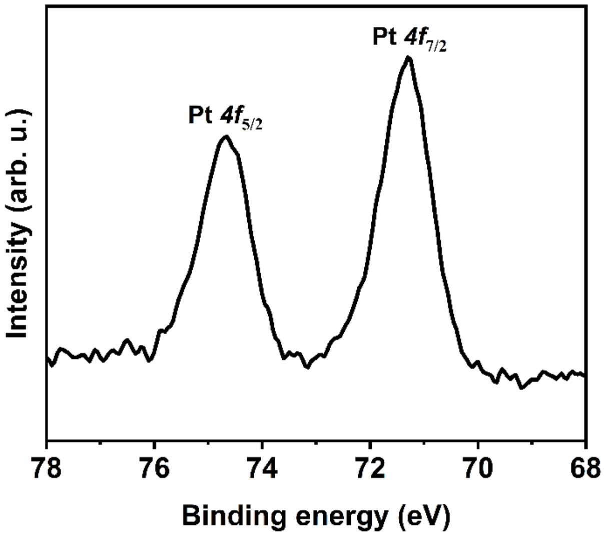

Supplementary Figure 5. The High-resolution XPS spectra of Pt *4f* of Pt_1Ag_{13}.

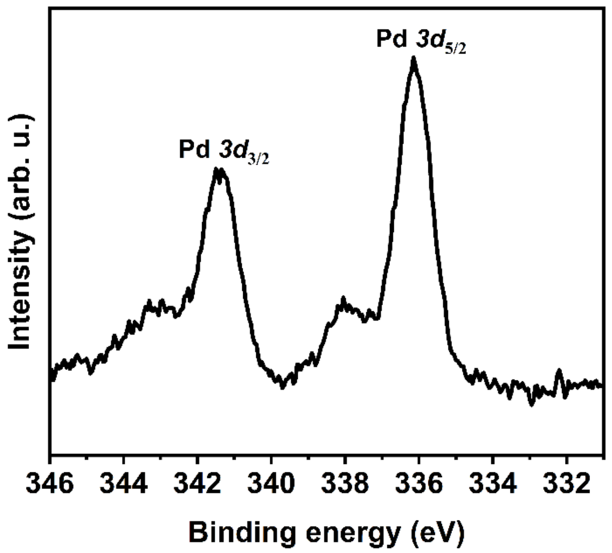

Supplementary Figure 6. The High-resolution XPS spectra of Pd *3d* of Pd_1Ag_{13}.

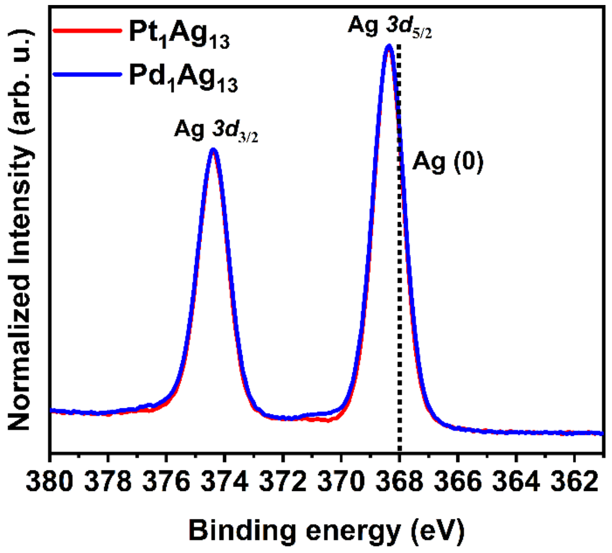

Supplementary Figure 7. The High-resolution XPS spectra of Ag *3d* of Pt_1Ag_{13} (red trace), and Pd_1Ag_{13} (blue trace).

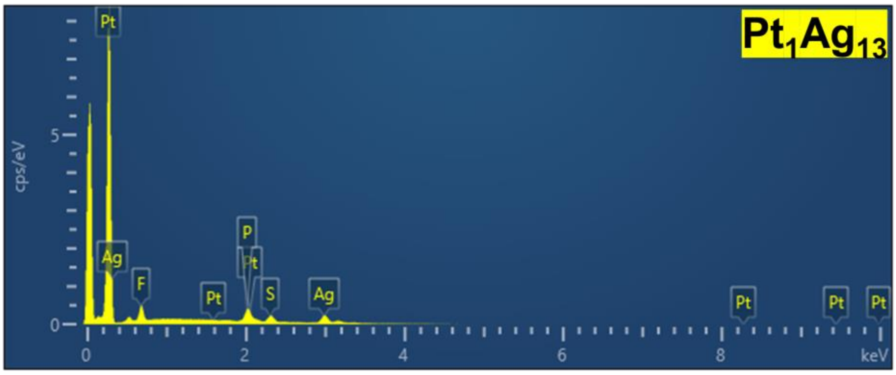

Supplementary Figure 8. EDS mapping of Pt_1Ag_{13}.

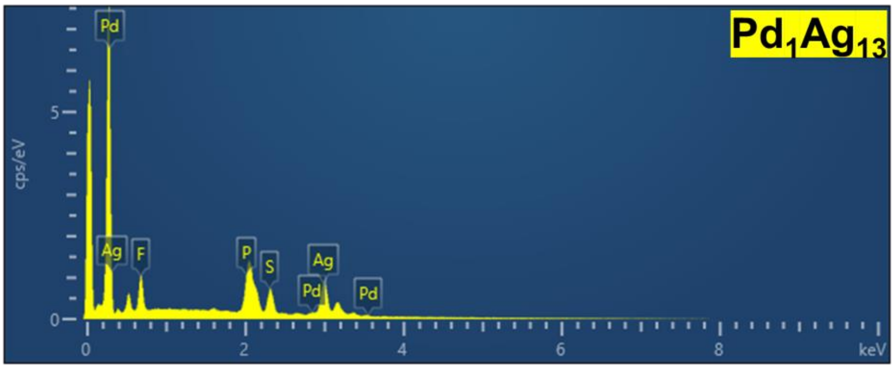

Supplementary Figure 9. EDS mapping of Pd_1Ag_{13}.

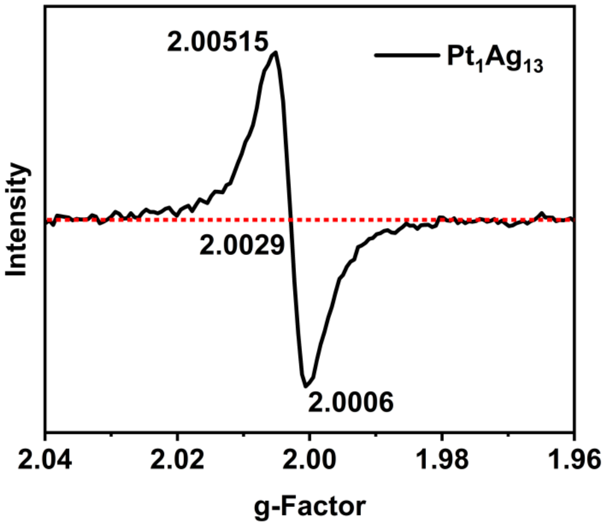

Supplementary Figure 10. EPR signals of Pt_1Ag_{13}.

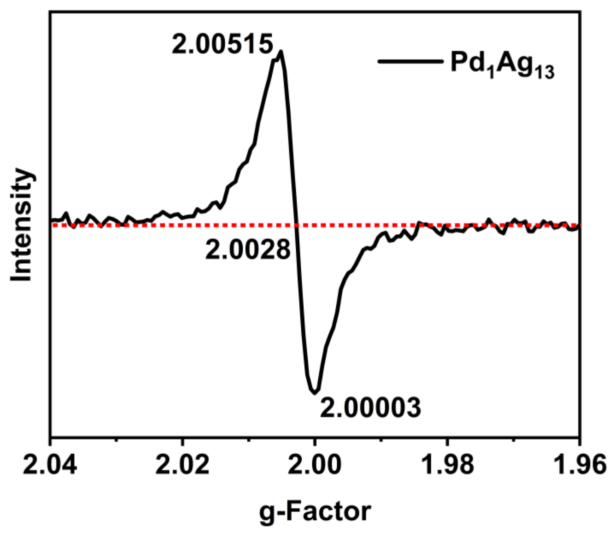

Supplementary Figure 11. EPR signals of Pd_1Ag_{13}.

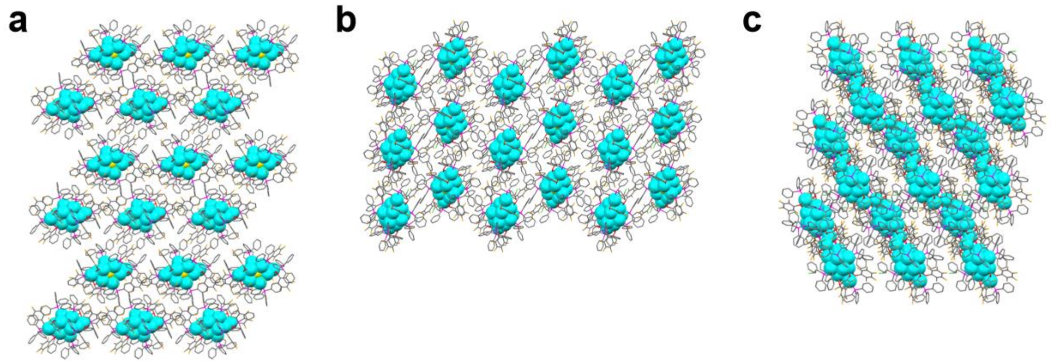

Supplementary Figure 12. The packing diagram of Pt_1Ag_{13}. View from (a) a direction, (b) b direction, and (c) c direction. Atom colors: yellow, Pt; sky blue, Ag; red, S; magenta, P; orange, F; gray, C. All hydrogen atoms are omitted for clarity.

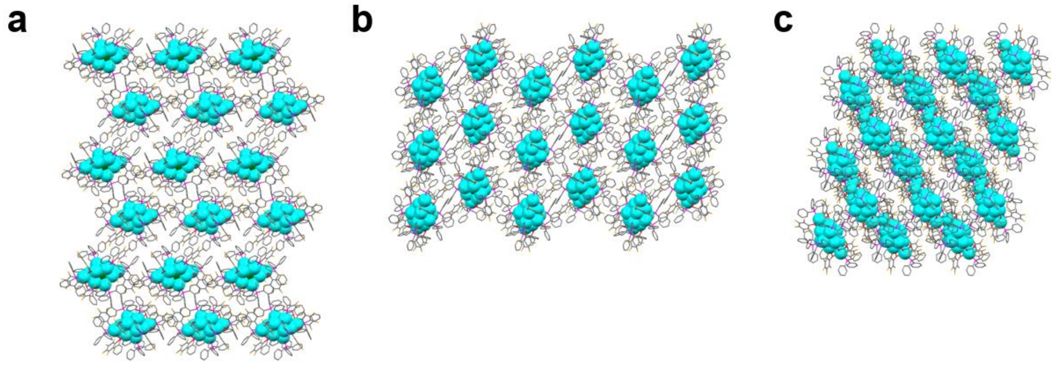

Supplementary Figure 13. The packing diagram of Pd_1Ag_{13}. View from (a) a direction, (b) b direction, and (c) c direction. Atom colors: green, Pd; sky blue, Ag; red, S; magenta, P; orange, F; gray, C. All hydrogen atoms are omitted for clarity.

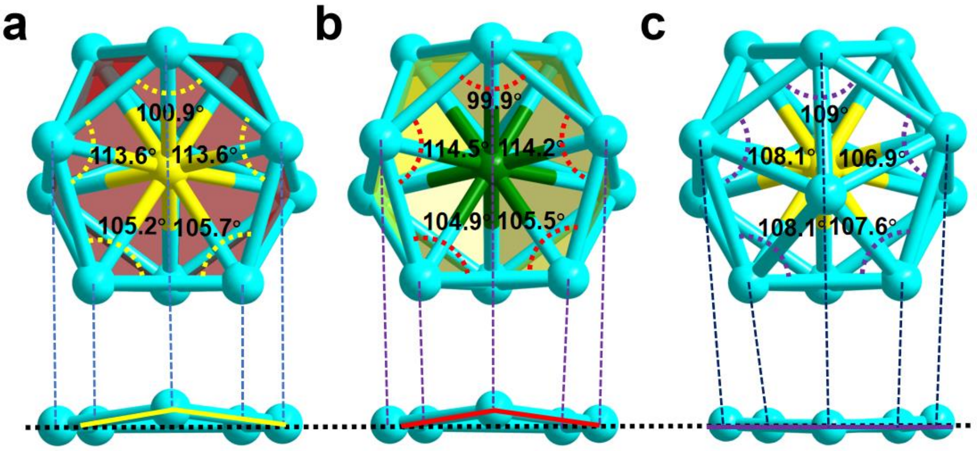

Supplementary Figure 14. The angle between Ag and Ag (∠Ag-Ag-Ag) of (a) Pt_1Ag_{13}, (b) Pd_1Ag_{13}, and (c) $Pt_1Ag_{14}(SR)_6(TPP)_8$ kernel. Color codes for atoms: yellow, Pt; green, Pd; sky blue, Ag.

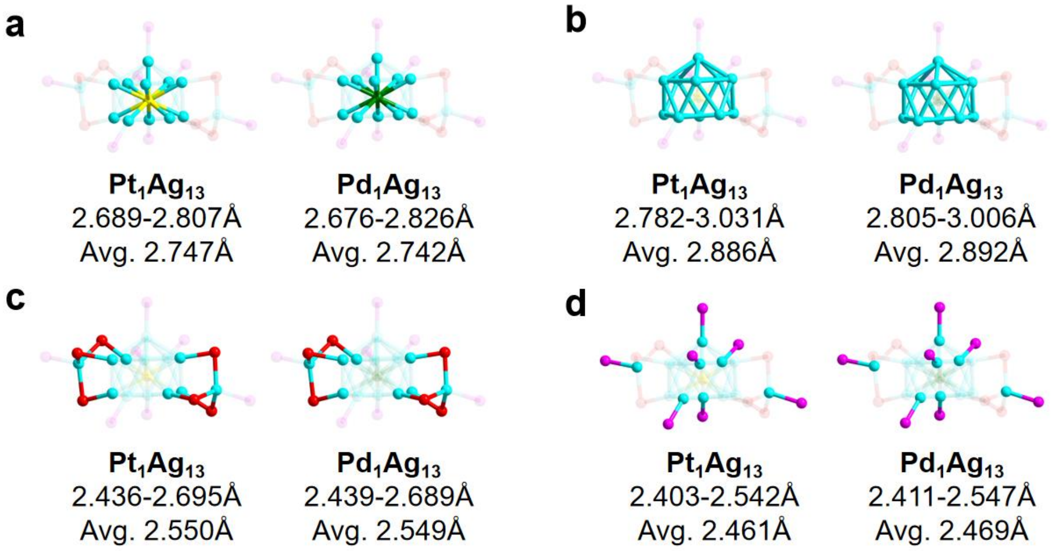

Supplementary Figure 15. Comparison of lengths of (a) M-Ag (M = Pt, Pd), (b) Ag-Ag, (c) Ag-S, and (d) Ag-P of Pt_1Ag_{13} and Pd_1Ag_{13}. The compared bonds are highlighted. Color code: yellow, Pt; green, Pd; sky blue, Ag; red, S; magenta, P.

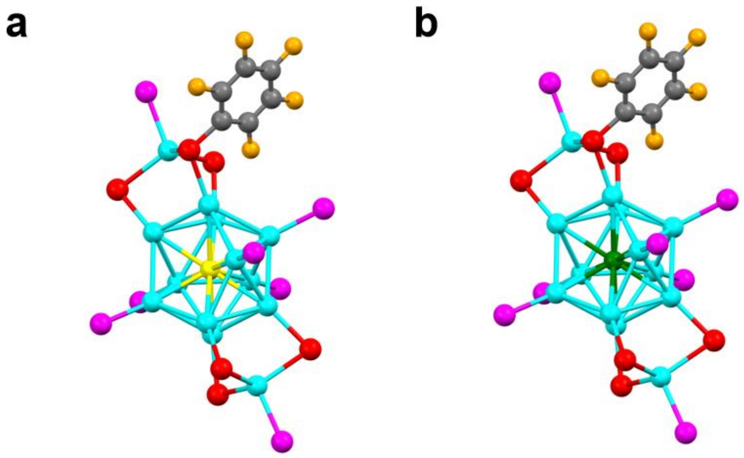

Supplementary Figure 16. The S-Ag connecting mode (six Ag_k-S-Ag_s) of (a) Pt_1Ag_{13} and (b) Pd_1Ag_{13} nanoclusters. Color code: yellow, Pt; green, Pd; sky blue, Ag; red, S; magenta, P; orange, F; gray, C. (Ag_k: the Ag atom in the kernel, Ag_s: the Ag atom in the shell).

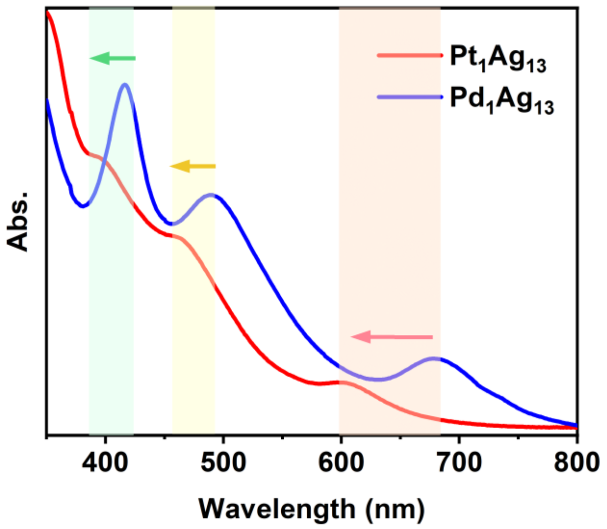

Supplementary Figure 17. UV-vis absorption spectra of Pt_1Ag_{13} and Pd_1Ag_{13} in the 2-Me-THF. The colored boxes represent the positions of the absorption peaks of Pt_1Ag_{13} and Pd_1Ag_{13}, while the arrow indicates that Pt_1Ag_{13} has undergone an overall blue shift relative to Pd_1Ag_{13}.

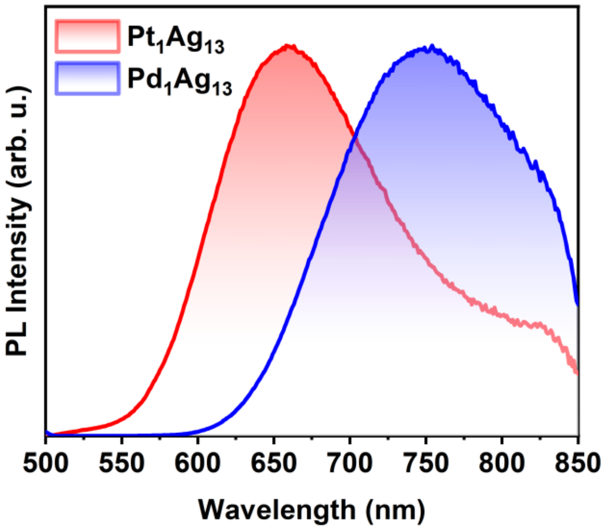

Supplementary Figure 18. PL spectra of Pt_1Ag_{13} and Pd_1Ag_{13} in 2-Me-THF solution.

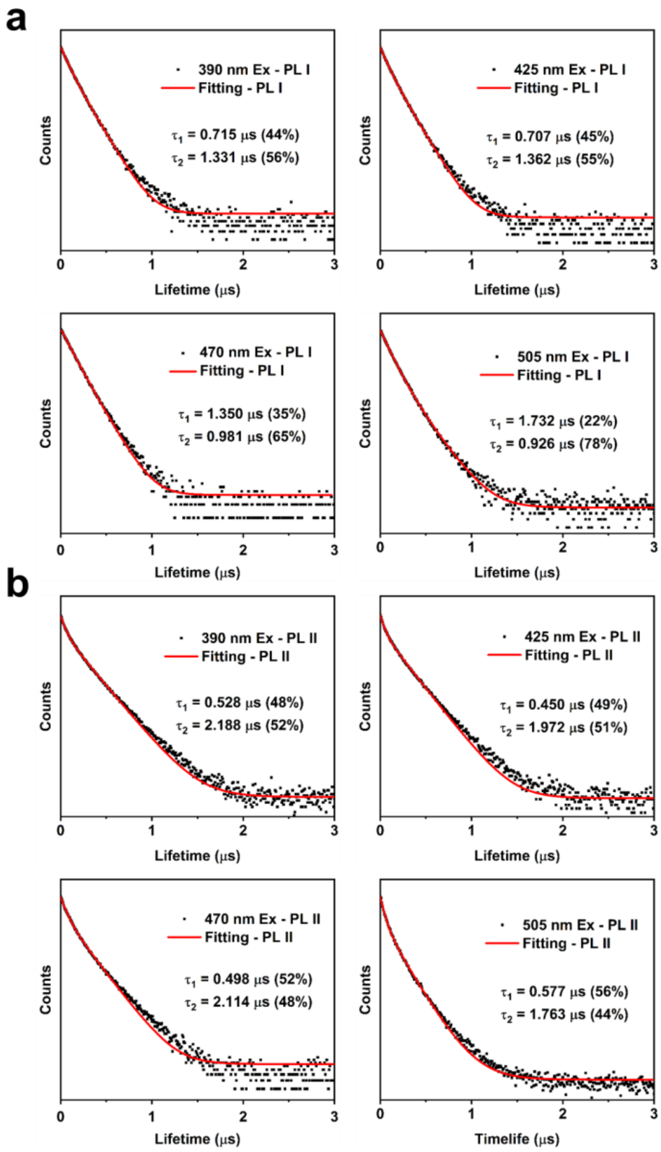

Supplementary Figure 19. PL decay curves and fitting curves of (a) PL I and (b) PL II of Pt_1Ag_{13} under different excitation wavelengths.

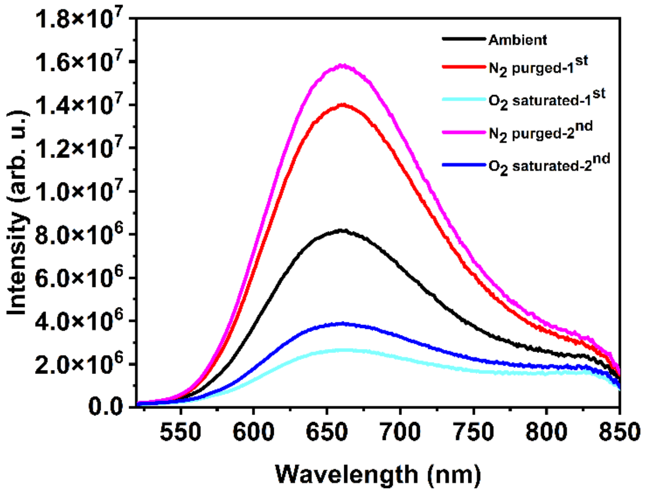

Supplementary Figure 20. PL spectra of Pt_1Ag_{13} in 2-Me-THF solution under ambient condition, N_2 purged, and O_2 saturated conditions, respectively.

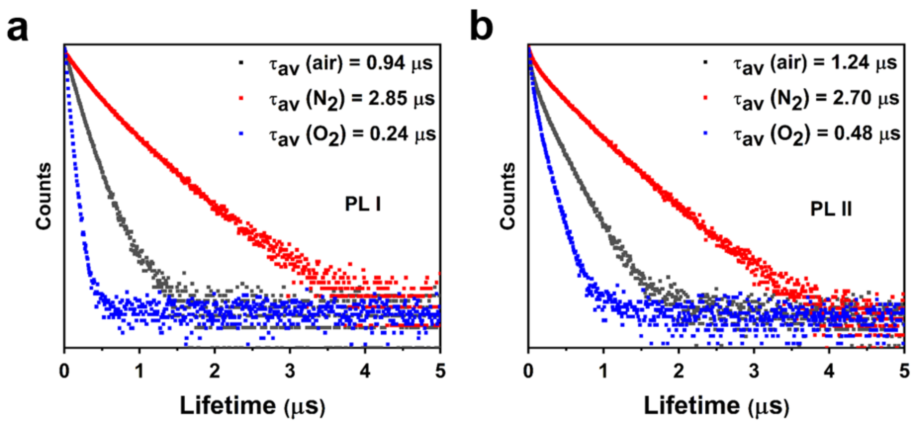

Supplementary Figure 21. PL decay profiles of Pt_1Ag_{13}. (a) PL I and (b) PL II decay profiles under ambient condition (black dots), N_2 purged (red dots), and O_2 saturated (blue dots) conditions.

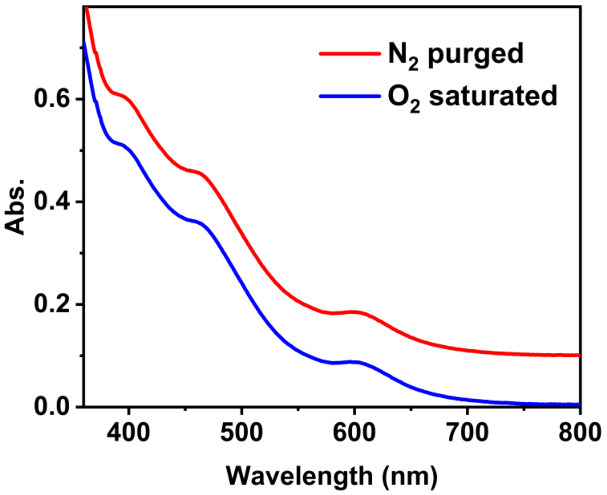

Supplementary Figure 22. UV-vis absorption spectra of Pt_1Ag_{13} in 2-Me-THF solution under N_2 purged (red) and O_2 saturated (blue) conditions.

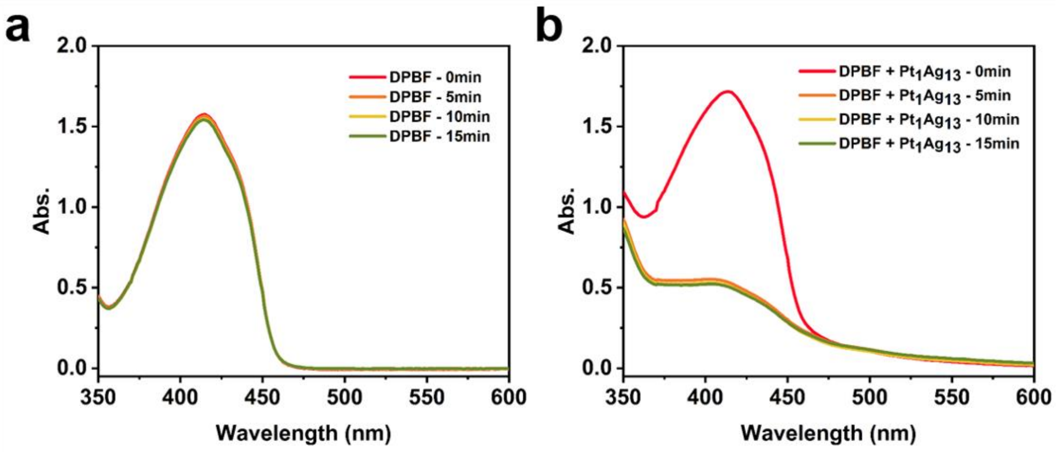

Supplementary Figure 23. Time-dependent UV-vis absorption spectra. (a) Time-dependent UV-vis absorption spectra of the 1, 3-diphenylisobenzofuran (DPBF) in 2-Me-THF solution. (b) Time-dependent UV-vis absorption spectra of the DPBF in 2-Me-THF solution mixed with Pt_1Ag_{13}.

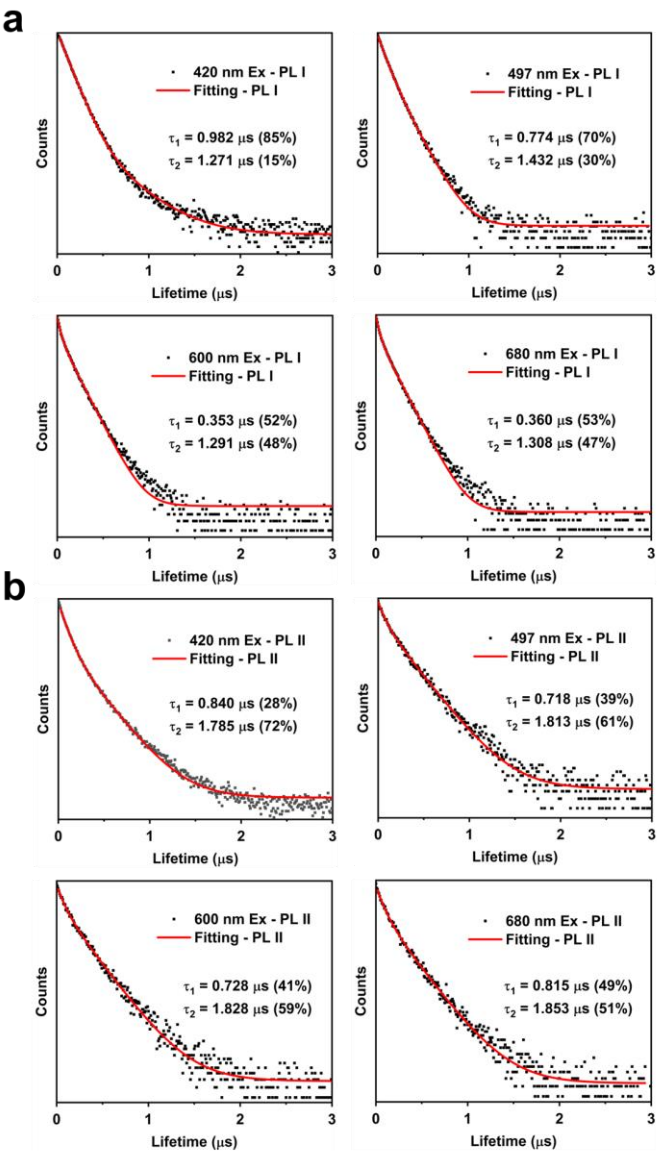

Supplementary Figure 24. PL decay curves and fitting curves of (a) PL I and (b) PL II of Pd_1Ag_{13} under different excitation wavelengths.

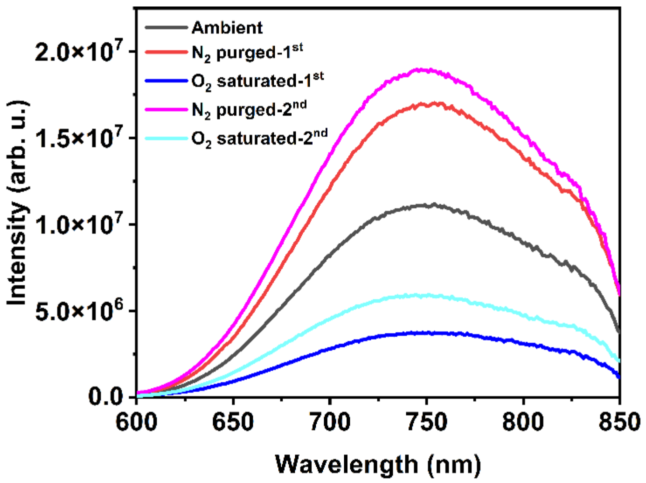

Supplementary Figure 25. PL spectra of Pd_1Ag_{13} in 2-Me-THF solution under ambient condition, N_2 purged, and O_2 saturated conditions, respectively.

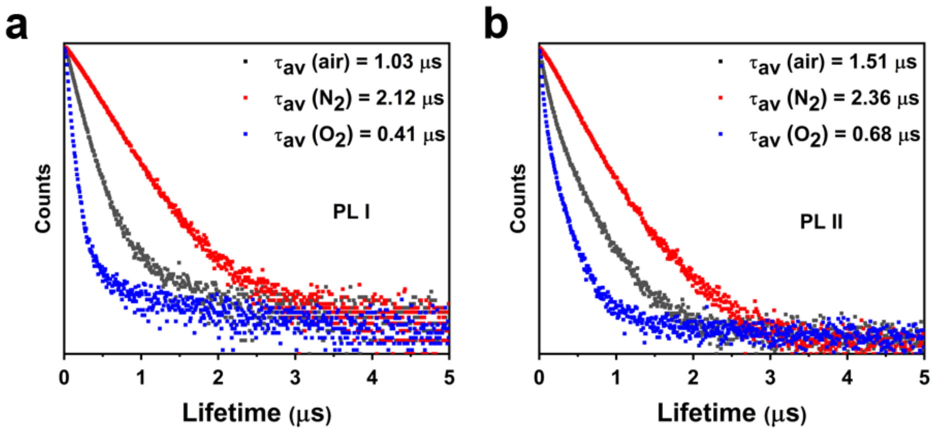

Supplementary Figure 26. PL decay profiles of Pd_1Ag_{13}. (a) PL I and (b) PL II decay profiles under ambient condition (black dots), N_2 purged (red dots), and O_2 saturated (blue dots) conditions.

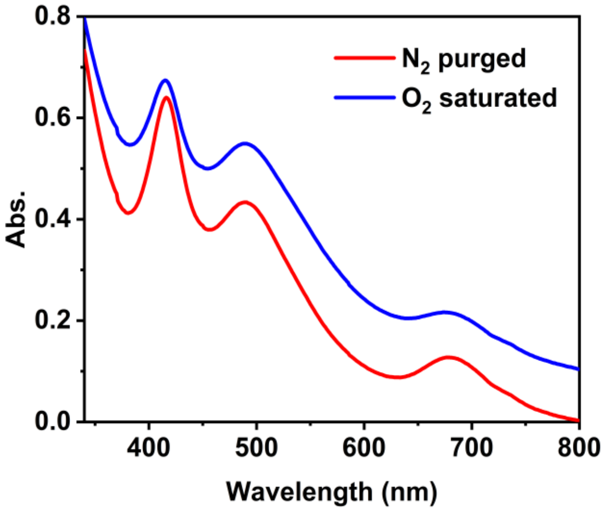

Supplementary Figure 27. UV-vis absorption spectra of Pd_1Ag_{13} in 2-Me-THF solution under N_2 purged (red) and O_2 saturated (blue) conditions.

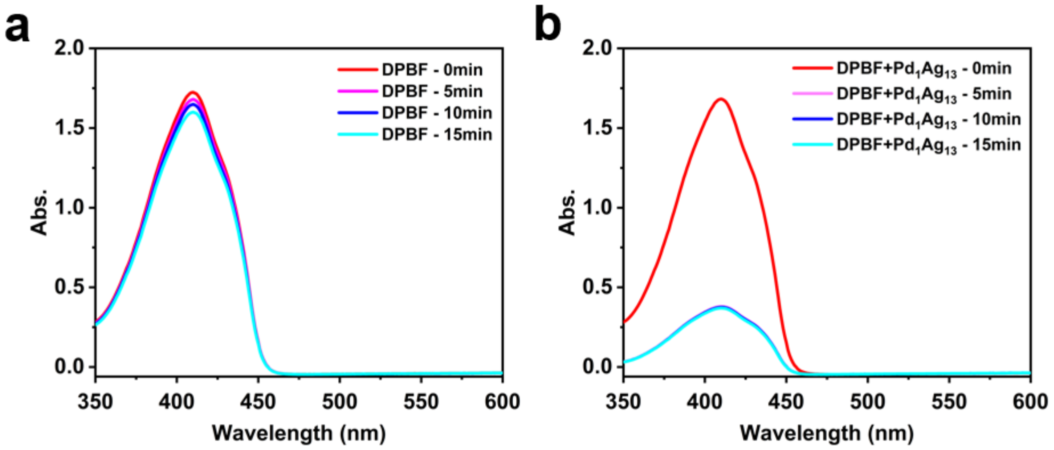

Supplementary Figure 28. Time-dependent UV-vis absorption spectra. (a) Time-dependent UV-vis absorption spectra of the 1, 3-diphenylisobenzofuran (DPBF) in 2-Me-THF solution. (b) Time-dependent UV-vis absorption spectra of the DPBF in 2-Me-THF solution mixed with Pd_1Ag_{13}.

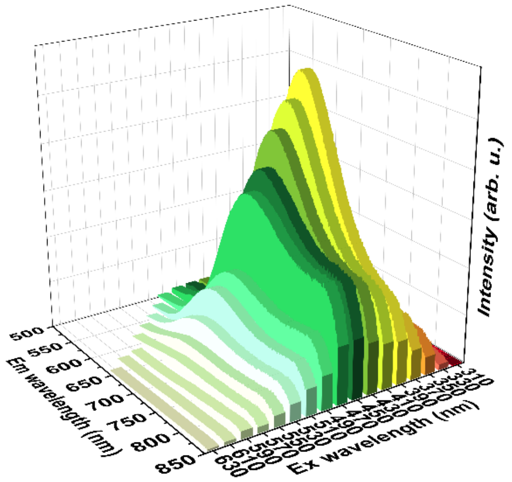

Supplementary Figure 29. The change trends of PL I and PL II upon different excitation of Pt_1Ag_{13} in the 2-Me-THF. PL I and PL II intensities were at a maximum at 390 and 470 nm, respectively.

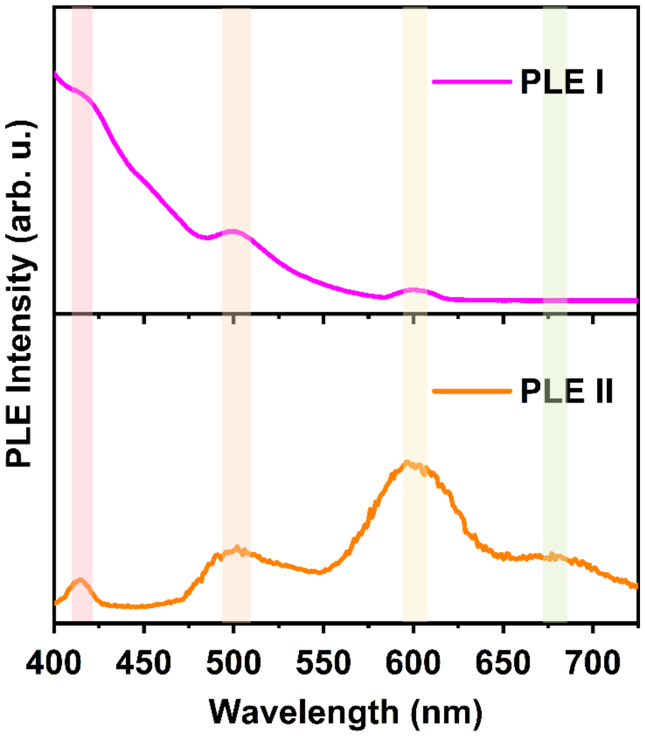

Supplementary Figure 30. PLE spectrum of Pd_1Ag_{13} at PL I and PL II wavelengths. The colored boxes indicate that PLE I and PLE II have identical excitation peaks at 420, 497, and 600 nm, with the difference being that PLE II has an additional excitation peak at 680 nm.

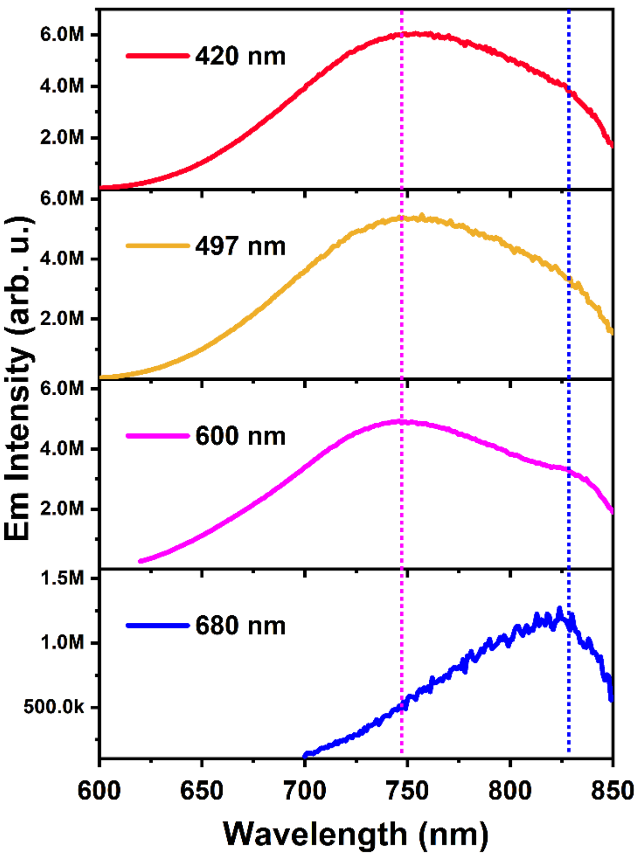

Supplementary Figure 31. PL spectra of Pd_1Ag_{13} in 2-Me-THF under excitation wavelengths of 420, 497, 600, and 680 nm, respectively. The pink dashed line represents PL I, and the blue dashed line represents PL II.

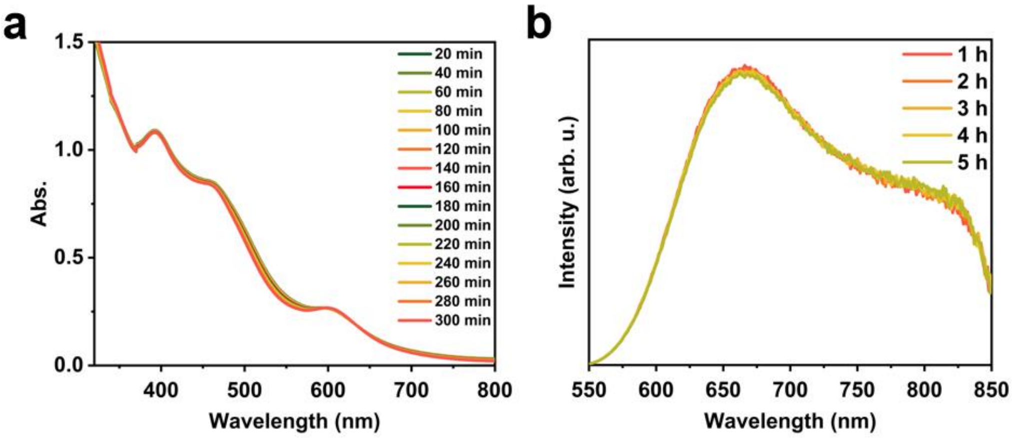

Supplementary Figure 32. Time-tracking (a) UV-vis and (b) PL spectra of Pt_1Ag_{13} in 2-Me-THF solution at room temperature.

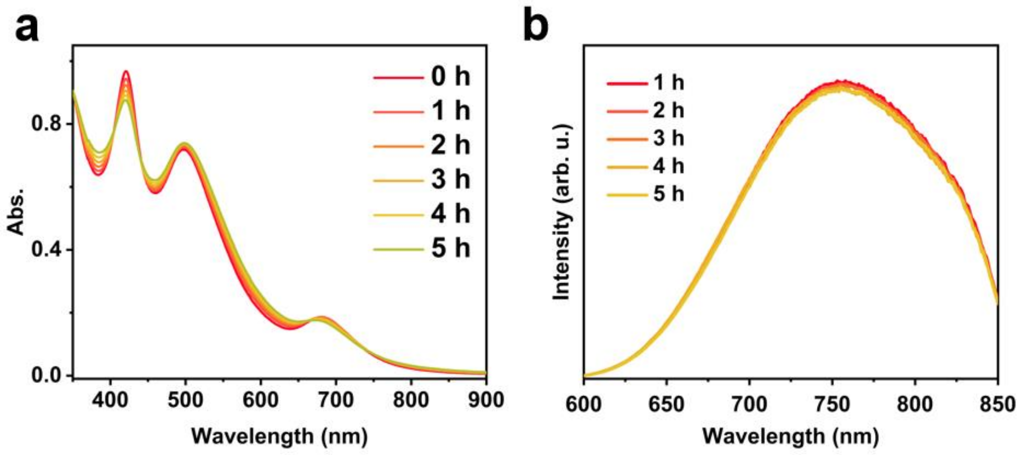

Supplementary Figure 33. Time-tracking (a) UV-vis and (b) PL spectra of Pd_1Ag_{13} in 2-Me-THF solution at room temperature.

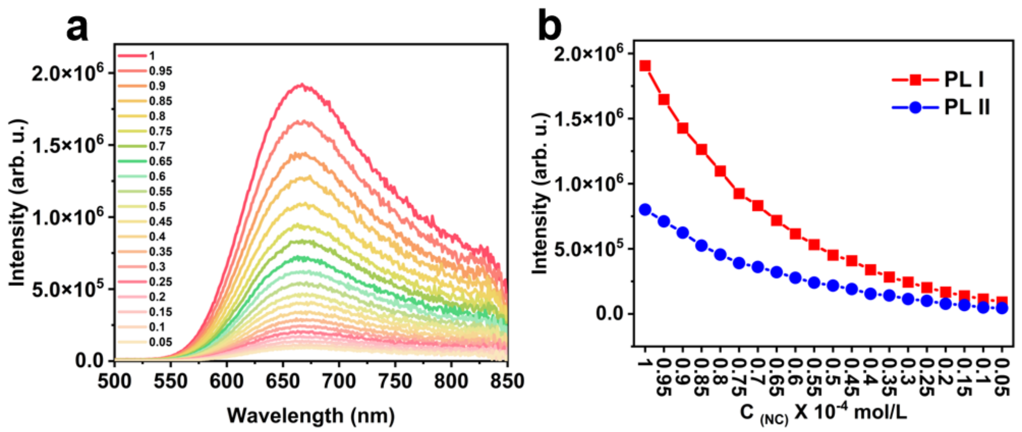

Supplementary Figure 34. (a) PL spectra of Pt_1Ag_{13} in 2-Me-THF solution at different concentrations. (b) The plotted intensity of PL I and PL II with different concentrations.

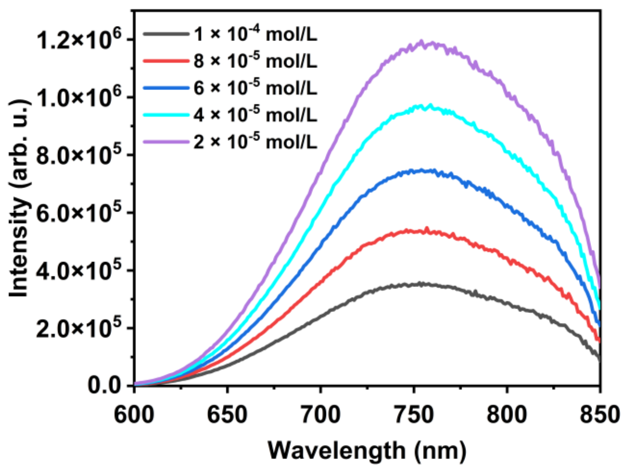

Supplementary Figure 35. PL spectra of Pd_1Ag_{13} in 2-Me-THF solution at different concentrations.

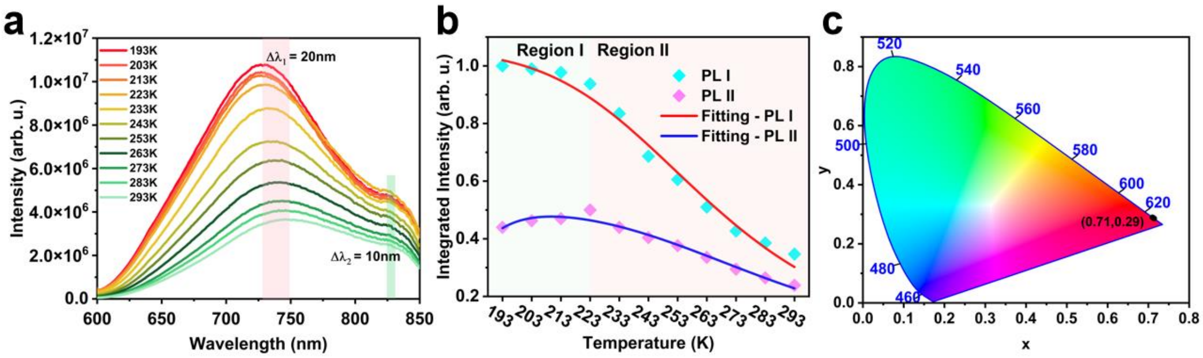

Supplementary Figure 36. Temperature-dependent PL spectra of Pd_1Ag_{13}. (a) Variable-temperature PL spectra of Pd_1Ag_{13} in 2-Me-THF. The color boxes represent the blue and red shift trends of PL I and PL II respectively. (b) Normalized integrated PL I and PL II intensities were fitted using Eqs 1 and 2, respectively; the integration of PL II is separated as regions I and II. The colored box on the left represents Region I, and the colored box on the right represents Region II. (c) CIE 1931 color space chromaticity diagram showing the luminescence color change of Pd_1Ag_{13} in the temperature range of 193-293 K.

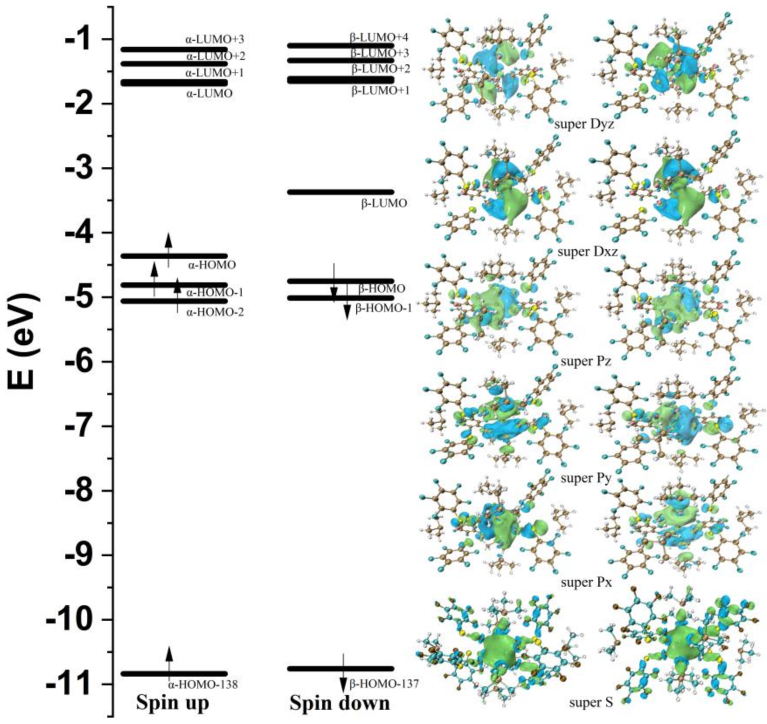

Supplementary Figure 37. Superatomic shell of Pt_1Ag_{13} core in its highest occupied molecular orbitals (HOMOs) and lowest unoccupied molecular orbitals (LUMOs).

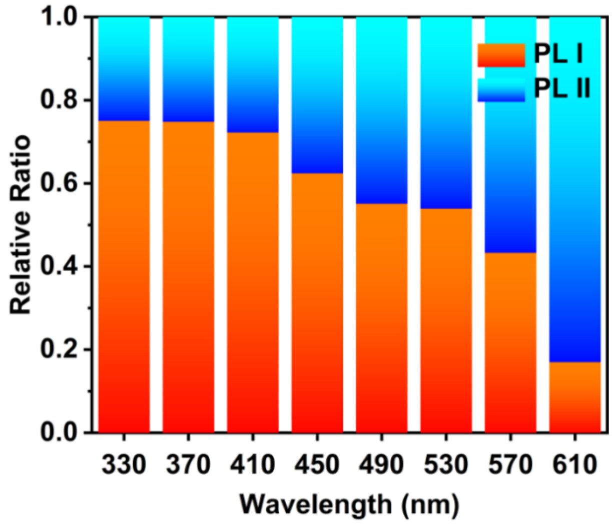

Supplementary Figure 38. PL intensity relative ratio of PL I and PL II of Pt_1Ag_{13} at different excitation.

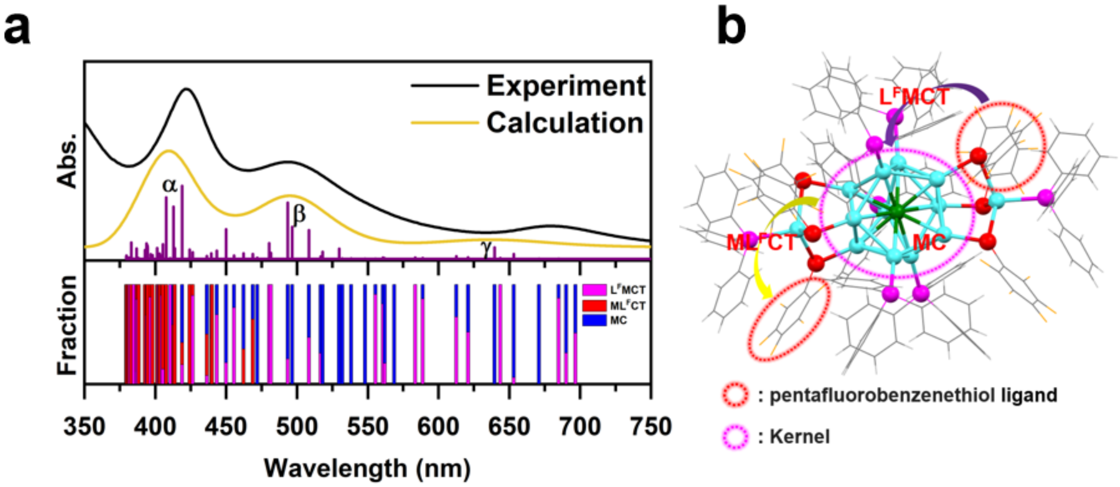

Supplementary Figure 39. DFT calculations of Pd_1Ag_{13}. (a) Experimental and calculated absorption spectrum of Pd_1Ag_{13} with contributions from metal-centered transition (MC), metal-ligand charge transfer (ML^FCT) and ligand-metal charge transfer (L^FMCT) excited states. (b) Intuitive diagram of charge transfer excited states.

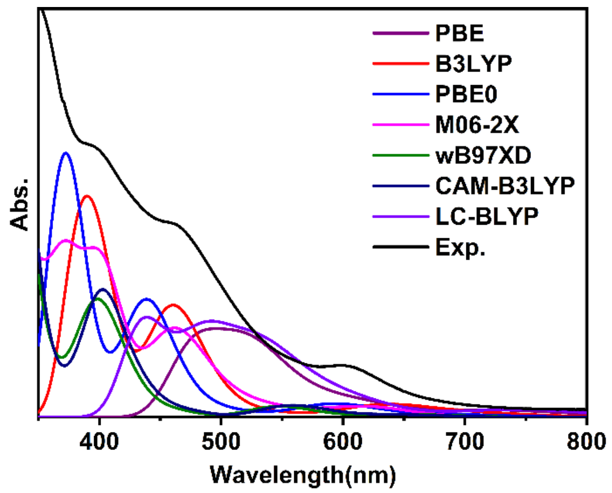

Supplementary Figure 40. Benchmark of different functionals (PBE, B3LYP, PBE0, M06-2X, LC-BLYP/ω = 0.01, CAM-B3LYP, ωB97XD) for TD-DFT calculation of liganded Pt_1Ag_{13} NC, experimental result of UV-vis spectrum is taken into comparison.

Supplementary Table 1. Atom ratios of Pt/Ag in $Pt_1Ag_{13}(PFBT)_6(TPP)_7$, and Pd/Ag in $Pd_1Ag_{13}(PFBT)_6(TPP)_7$ nanoclusters were calculated from inductively coupled plasma-atomic emission spectroscopy (ICP-AES) and X-ray photoelectric spectroscopy (XPS).

**$Pt_1Ag_{13}(PFBT)_6(TPP)_7$**	**Pt atom**	**Ag atom**
ICP Experimental Ratio	7.18 %	92.82 %
XPS Experimental Ratio	7.59 %	92.41 %
Theoretical Ratio	7.14 %	92.86 %
**$Pd_1Ag_{13}(PFBT)_6(TPP)_7$**	**Pd atom**	**Ag atom**
ICP Experimental Ratio	7.15 %	92.85 %
XPS Experimental Ratio	7.39 %	92.61 %
Theoretical Ratio	7.14 %	92.86 %

Supplementary Table 2. Crystal data and structure refinement for the Pt_1Ag_{13} nanocluster. The CCDC number of $Pt_1Ag_{13}(PFBT)_6(TPP)_7$ is 2300261.

**$Pt_1Ag_{13}(PFBT)_6(TPP)_7$**	
Empirical formula	$C_{164}H_{109}Ag_{13}Cl_4F_{30}P_7Pt_1S_6$
Formula weight	4797.85

Temperature/K	120
Crystal system	triclinic
Space group	P-1
a/Å	16.4044(18)
b/Å	18.529(2)
c/Å	27.185(3)
α/°	90.503(9)
β/°	92.837(9)
γ/°	100.307(8)
Volume/$Å^3$	8118.5(15)
Z	2
$\rho_{calc}g/cm^3$	1.963
μ/mm^{-1}	16.547
F(000)	4642.0
Radiation	CuKα (λ = 1.54186)
2θ range for data collection/°	11.78 to 139.52
Index ranges	-19 ≤ h ≤ 18, -22 ≤ k ≤ 21, -33 ≤ l ≤ 22
Reflections collected	74769
Independent reflections	29007 [R_{int} = 0.0388, R_{sigma} = 0.0545]
Data/restraints/parameters	29007/42/2026
Goodness-of-fit on F^2	0.956
Final R indexes [I>=2σ (I)]	R_1 = 0.0436, wR_2 = 0.1090
Final R indexes [all data]	R_1 = 0.0549, wR_2 = 0.1136
Largest diff. peak/hole / e $Å^{-3}$	5.00/-2.76

Supplementary Table 3. Crystal data and structure refinement for the Pd_1Ag_{13} nanocluster. The CCDC number of $Pd_1Ag_{13}(PFBT)_6(TPP)_7$ is 2304388.

$Pd_1Ag_{13}(PFBT)_6(TPP)_7$	
Empirical formula	$C_{164}H_{109}Ag_{13}Cl_4F_{30}P_7Pd_1S_6$
Formula weight	4708.36
Temperature/K	120
Crystal system	triclinic
Space group	P-1
a/Å	16.3816(18)
b/Å	18.545(2)
c/Å	27.157(3)
α/°	90.510(9)
β/°	92.755(9)
γ/°	100.269(9)
Volume/Å^3	8107.5(16)
Z	2
ρ_{calc}g/cm^3	1.929
μ/mm^{-1}	15.874
F(000)	4576.0
Radiation	CuKα (λ = 1.54186)
2θ range for data collection/°	6.64 to 139.606
Index ranges	-18 ≤ h ≤ 19, -11 ≤ k ≤ 22, -33 ≤ l ≤ 30
Reflections collected	68046
Independent reflections	29059 [R_{int} = 0.0255, R_{sigma} = 0.0257]
Data/restraints/parameters	29059/1194/2035
Goodness-of-fit on F^2	1.041
Final R indexes [I>=2σ (I)]	R_1 = 0.0429, wR_2 = 0.1166
Final R indexes [all data]	R_1 = 0.0467, wR_2 = 0.1196

Largest diff. peak/hole / e Å^{-3}	3.18/-1.53

Supplementary Table 4. The lifetime and relative amplitude of Pt_1Ag_{13} in 2-Me-THF under different excitation wavelengths.

Ex.	PL I (660 nm)					PL II (825 nm)				
	τ_1 (μs)	τ_1 (%)	τ_2 (μs)	τ_2 (%)	τ_{av} (μs)	τ_1 (μs)	τ_1 (%)	τ_2 (μs)	τ_2 (%)	τ_{av} (μs)
390 nm	0.715	44	1.331	56	1.148	0.528	48	2.188	52	1.886
425 nm	0.707	45	1.362	55	1.167	0.450	49	1.972	51	1.698
470 nm	1.350	35	0.981	65	1.138	0.498	52	2.114	48	1.785
505 nm	1.732	22	0.926	78	1.204	0.577	56	1.763	44	1.414

Supplementary Table 5. The lifetime and relative amplitude of Pd_1Ag_{13} in 2-Me-THF under different excitation wavelengths.

Ex.	PL I (748 nm)					PL II (830 nm)				
	τ_1 (μs)	τ_1 (%)	τ_2 (μs)	τ_2 (%)	τ_{av} (μs)	τ_1 (μs)	τ_1 (%)	τ_2 (μs)	τ_2 (%)	τ_{av} (μs)
420 nm	0.982	85	1.271	15	1.034	0.840	28	1.785	72	1.509
497 nm	0.774	70	1.432	30	1.059	0.718	39	1.813	61	1.592
600 nm	0.353	52	1.291	48	1.076	0.728	41	1.828	59	1.590
680 nm	0.360	53	1.308	47	1.085	0.815	49	1.853	51	1.545

Supplementary Table 6. The PL peak intensity of Pt_1Ag_{13} in 2-Me-THF in the range of 193-293 K.

Temperature	PL I peak intensity	PL II peak intensity
193 K	8063810	576519
203 K	7925847	597804
213 K	7797159	604944
223 K	7384784	632089
233 K	6976262	673509
243 K	6225481	710139
253 K	5204553	758401
263 K	4058429	805824
273 K	2998106	758133
283 K	2668034	685726

293 K	2212373	571623

Supplementary Table 7. Fitting parameter obtained by fitting the temperature dependence of the PL I and PL II intensities of Pt_1Ag_{13} with Arrhenius equations, taking into account one (PL I) and two (PL II) non-radiative channels.

Peak	a_1	Ea_1(meV)	a_2	Ea_2(meV)
PL I	160.26	48.94	NA	NA
PL II	39.56	104.47	-8.54	37.62

Supplementary Table 8. The PL peak intensity of Pd_1Ag_{13} in 2-Me-THF in the range of 193-293 K.

Temperature	PL I peak intensity	PL II peak intensity
193 K	10517528	4388509
203 K	10404407	4619359
213 K	10275935	4691043
223 K	9857195	4992006
233 K	8775966	4389808
243 K	7218363	4035611
253 K	6367191	3755102
263 K	5363243	3350602
273 K	4490583	2939645
283 K	4052640	2639093
293 K	3648783	2384422

Supplementary Table 9. Fitting parameter obtained by fitting the temperature dependence of the PL I and PL II intensities of Pd_1Ag_{13} with Arrhenius equations, taking into account one (PL I) and two (PL II) non-radiative channels.

Peak	a_1	Ea_1(meV)	a_2	Ea_2(meV)
PL I	115.73	20.81	NA	NA
PL II	58.27	136.62	-10.43	19.28

Supplementary Table 10. Selected orbitals of Pt_1Ag_{13}.

α-HOMO-2 (-5.063 eV)<br>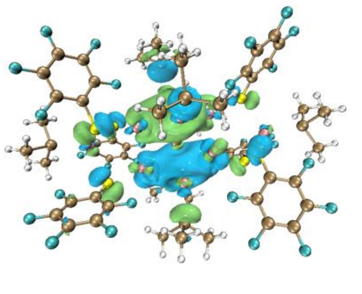	α-HOMO-1 (-4.805 eV)<br>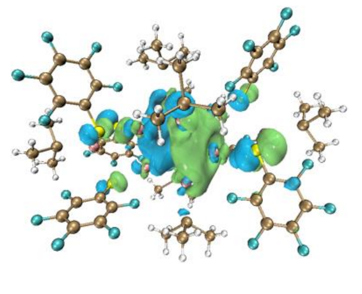	α-HOMO (-4.361 eV)<br>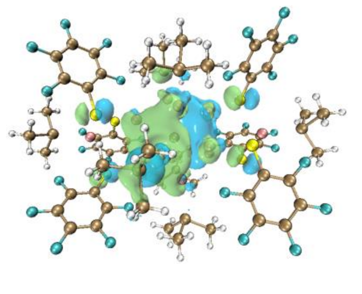
α-LUMO (-1.694 eV)<br>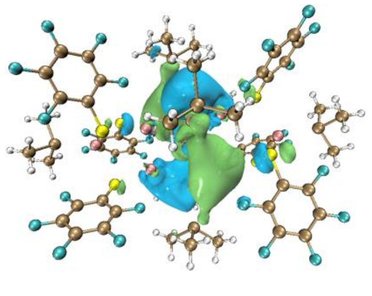	α-LUMO+1 (-1.661 eV)<br>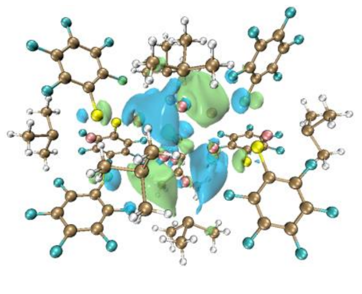	α-LUMO+2 (-1.380 eV)<br>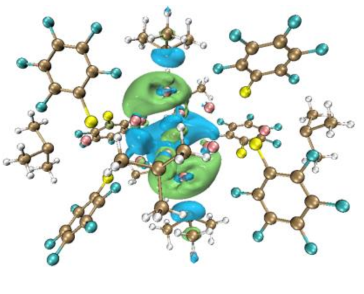
α-LUMO+3 (-1.160 eV)<br>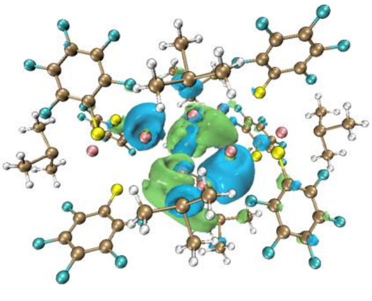	α-LUMO+4 (-1.070 eV)<br>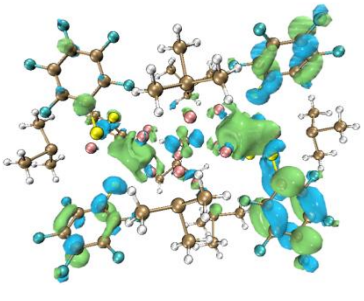	α-LUMO+5 (-0.862 eV)<br>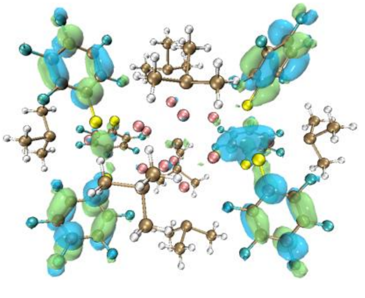
α-LUMO+6 (-0.705 eV)<br>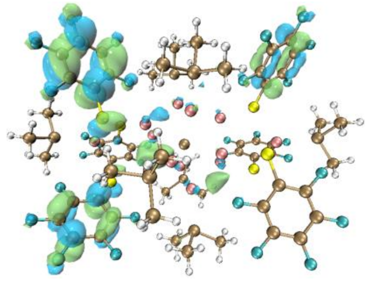	α-LUMO+7 (-0.632 eV)<br>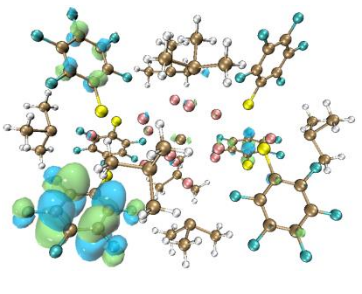	α-LUMO+8 (-0.613 eV)<br>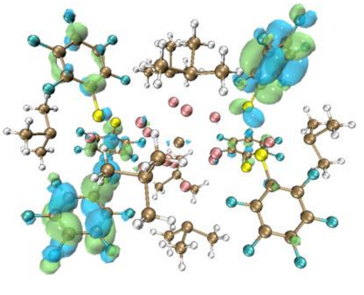
α-LUMO+9 (-0.588 eV)<br>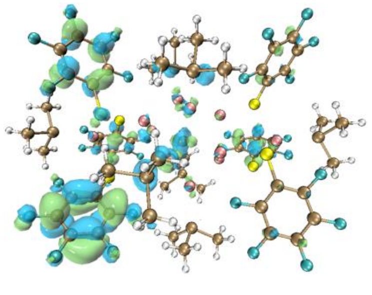	α-LUMO+10 (-0.548 eV)<br>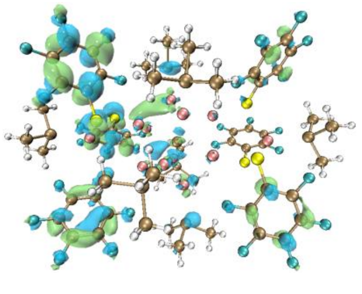	α-LUMO+11 (-0.524 eV)<br>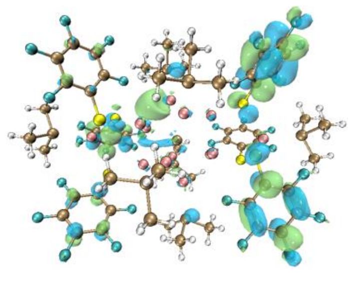

α-LUMO+12 (-0.505 eV)<br>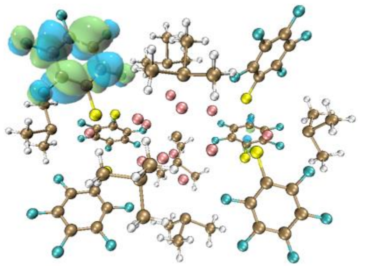	α-LUMO+13 (-0.465 eV)<br>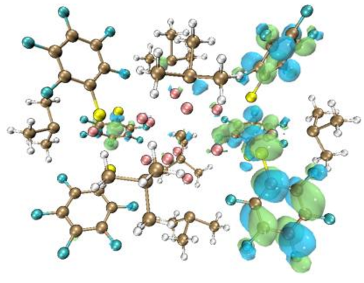	α-LUMO+14 (-0.445 eV)<br>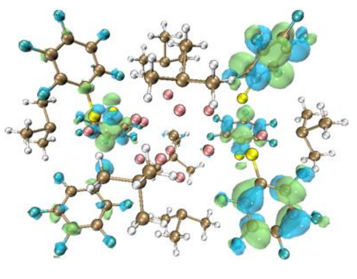
α-LUMO+15 (-0.437 eV)<br>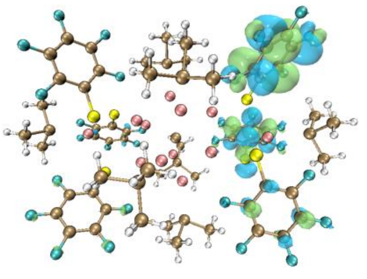	α-LUMO+16 (-0.415 eV)<br>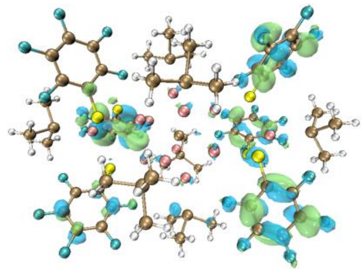	α-LUMO+17 (-0.397 eV)<br>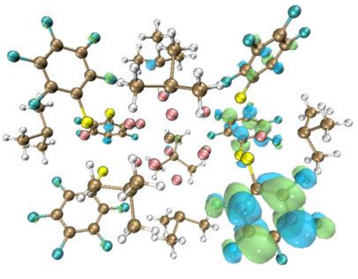
α-LUMO+18 (-0.380 eV)<br>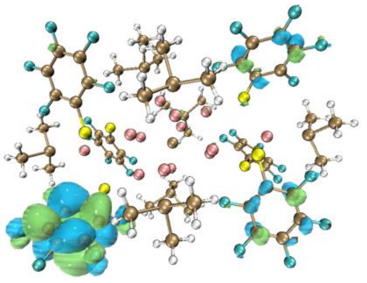	α-LUMO+19 (-0.306 eV)<br>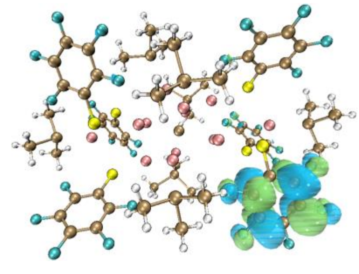	β-HOMO-28 (-7.264 eV)<br>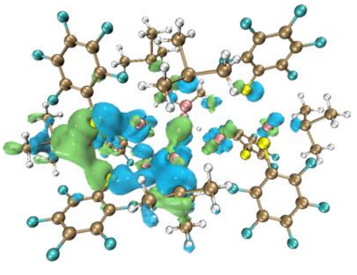
β-HOMO-22 (-6.939 eV)<br>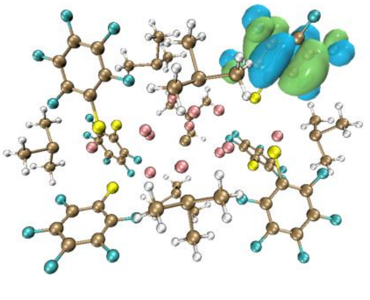	β-HOMO-21 (-6.919 eV)<br>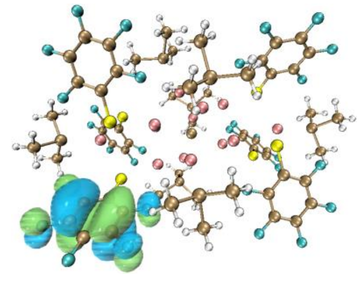	β-HOMO-20 (-6.845 eV)<br>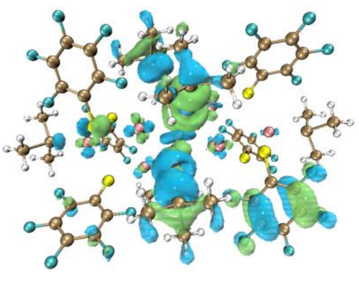
β-HOMO-19 (-6.837 eV)<br>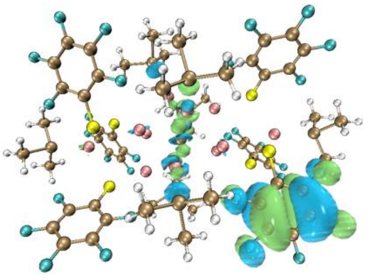	β-HOMO-18 (-6.657 eV)<br>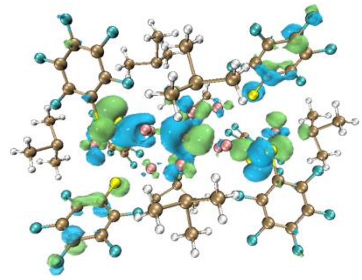	β-HOMO-17 (-6.542 eV)<br>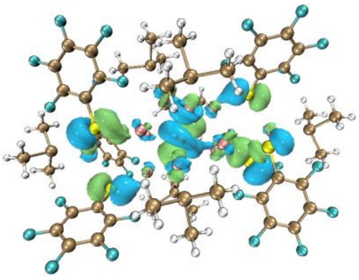

β-HOMO-16 (-6.460 eV)<br>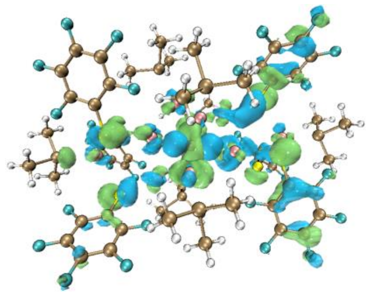	β-HOMO-15 (-6.420 eV)<br>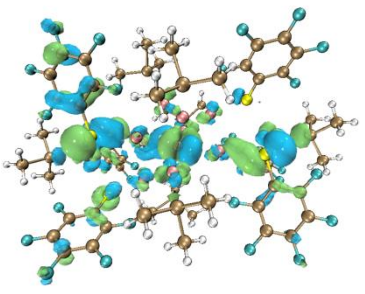	β-HOMO-14 (-6.313 eV)<br>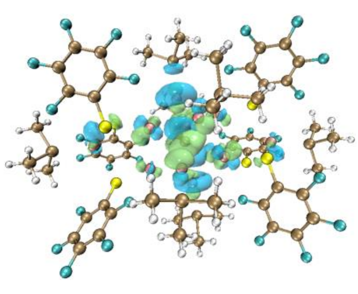
β-HOMO-13 (-6.291 eV)<br>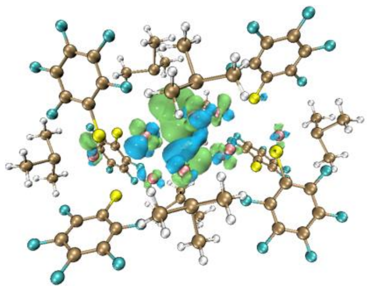	β-HOMO-12 (-6.187 eV)<br>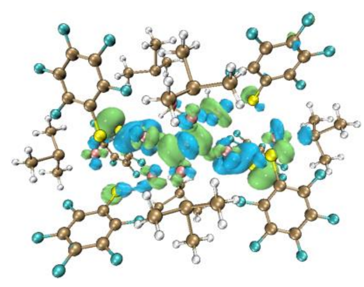	β-HOMO-11 (-6.095 eV)<br>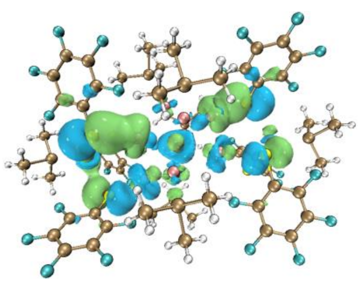
β-HOMO-10 (-6.066 eV)<br>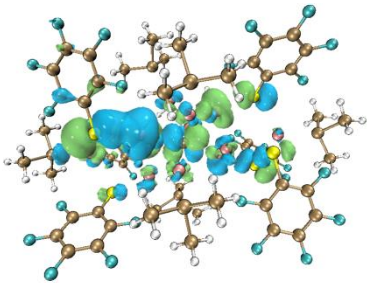	β-HOMO-9 (-6.019 eV)<br>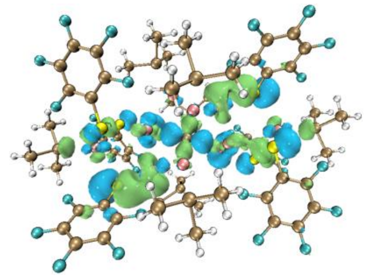	β-HOMO-8 (-5.984 eV)<br>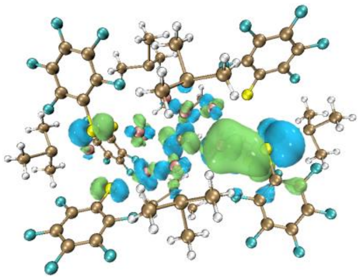
β-HOMO-7 (-5.686 eV)<br>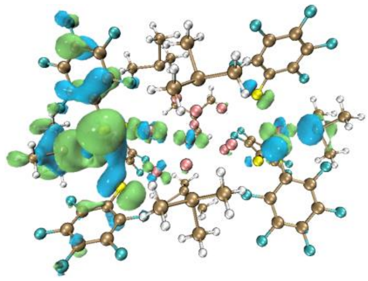	β-HOMO-6 (-5.571 eV)<br>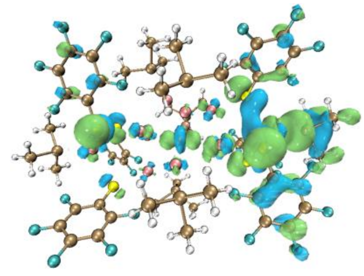	β-HOMO-5 (-5.485 eV)<br>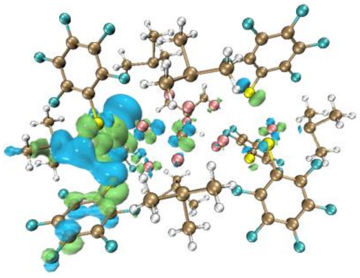
β-HOMO-4 (-5.460 eV)<br>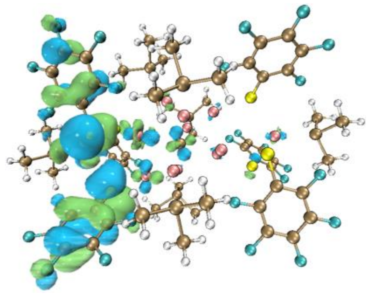	β-HOMO-3 (-5.422 eV)<br>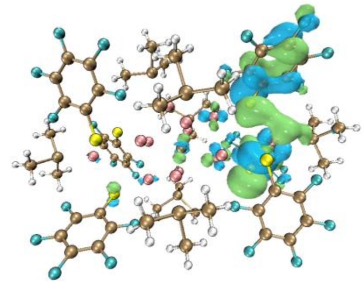	β-HOMO-2 (-5.362 eV)<br>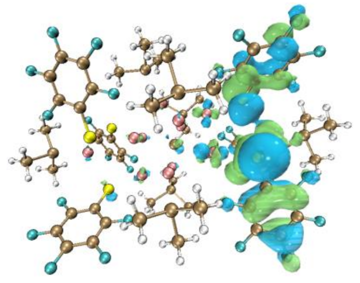

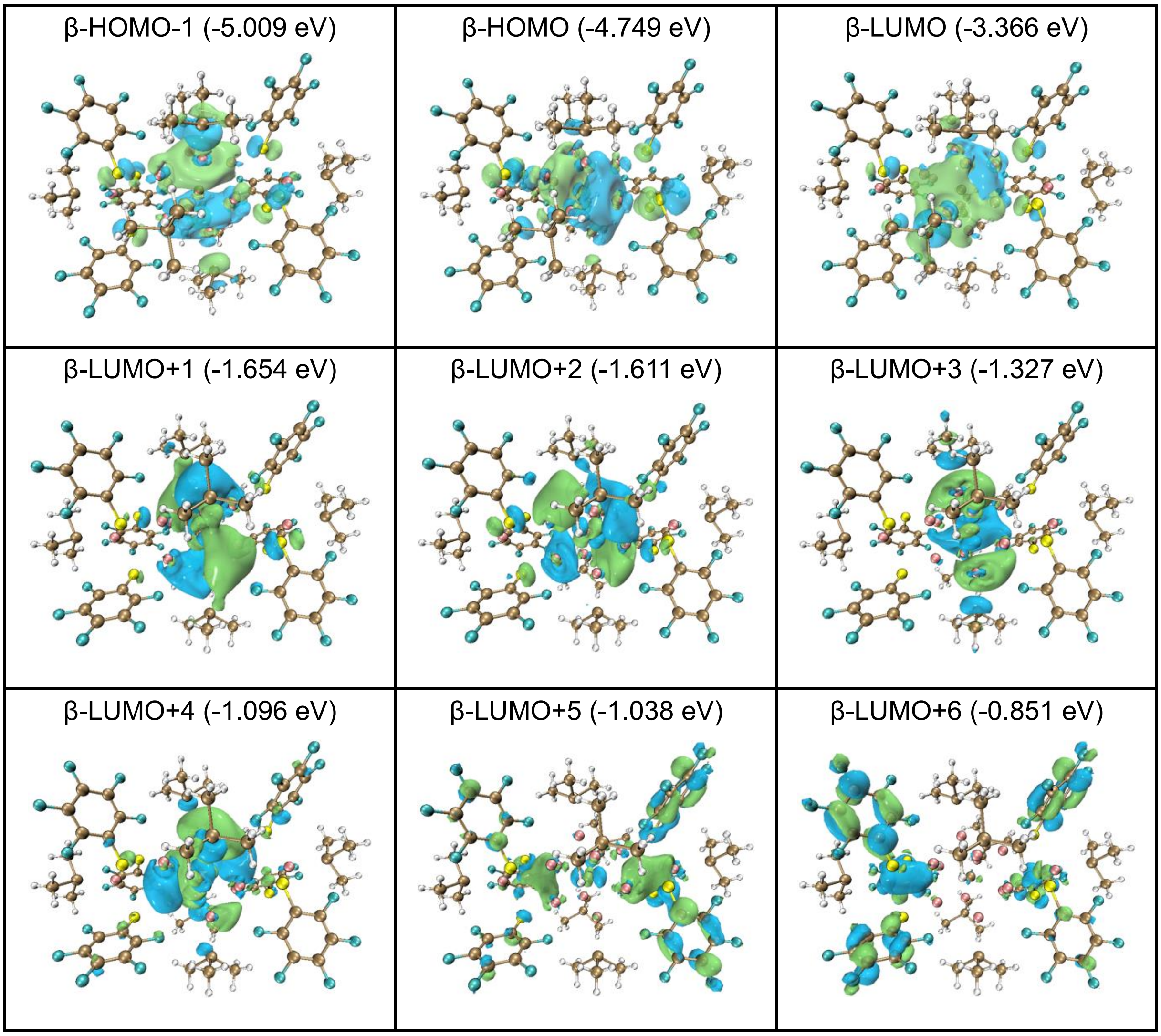

Supplementary Table 11. The selected excited states, energy, oscillator strength, and the most probable transitions of Pt_1Ag_{13} from TD-DFT calculations.

State	Energy (eV)	Energy (nm)	Oscillator strength (a.u.)	Most probable transitions	Weight of Transition	Nature of major transitions
1	1.9125	648.28	0.0024	β-HOMO-3→β-LUMO	0.311	L^{F}MCT
				α-HOMO→α-LUMO	0.228	MC
				β-HOMO-9→β-LUMO	0.21	MC
2	1.9300	642.41	0.0189	α-HOMO→α-LUMO	0.737	MC
3	1.9549	634.22	0.0234	α-HOMO→α-LUMO+1	0.772	MC
4	1.9713	628.95	0.0055	β-HOMO-2→β-LUMO	0.173	L^{F}MCT
				β-HOMO-8→β-LUMO	0.162	MC
				α-HOMO→α-LUMO+1	0.143	MC
5	2.0409	607.5	0.0005	β-HOMO-10→β-LUMO	0.276	MC
				β-HOMO-4→β-LUMO	0.265	L^{F}MCT
				β-HOMO-12→β-LUMO	0.143	MC

				β-HOMO-8→β-LUMO	0.076	MC
6	2.1264	583.07	0.0055	β-HOMO-7→β-LUMO	0.832	L^{F}MCT
				β-HOMO-10→β-LUMO	0.054	MC
7	2.2058	562.08	0.0031	β-HOMO-13→β-LUMO	0.603	MC
				β-HOMO-6→β-LUMO	0.14	L^{F}MCT
8	2.2889	541.68	0.0006	β-HOMO-12→β-LUMO	0.515	MC
				β-HOMO-10→β-LUMO	0.33	MC
9	2.3403	529.78	0.0095	α-HOMO→α-LUMO+2	0.493	MC
				α-HOMO→α-LUMO+3	0.226	MC
				β-HOMO→β-LUMO+1	0.148	MC
10	2.3496	527.68	0.0038	β-HOMO-14→β-LUMO	0.793	MC
11	2.3593	525.51	0.0041	α-HOMO-1→α-LUMO	0.411	MC
				β-HOMO→β-LUMO+1	0.342	MC
12	2.3969	517.27	0.0006	β-HOMO→β-LUMO+2	0.477	MC
				α-HOMO-1→α-LUMO+1	0.436	MC
13	2.4813	499.67	0.0118	α-HOMO→α-LUMO+4	0.484	ML^{F}CT
				β-HOMO-1→β-LUMO+1	0.202	MC
14	2.4896	498.01	0.0079	α-HOMO-2→α-LUMO	0.311	MC
				β-HOMO-1→β-LUMO+1	0.287	MC
				α-HOMO→α-LUMO+4	0.209	ML^{F}CT
15	2.5153	492.92	0.0018	α-HOMO→α-LUMO+3	0.531	MC
				α-HOMO→α-LUMO+2	0.251	MC
16	2.5203	491.94	0.0121	β-HOMO-15→β-LUMO	0.235	L^{F}MCT
				α-HOMO-1→α-LUMO	0.187	MC
				β-HOMO→β-LUMO+1	0.186	MC
17	2.5348	489.13	0.0036	α-HOMO-2→α-LUMO+1	0.405	MC
				β-HOMO-1→β-LUMO+2	0.335	MC
18	2.5500	486.21	0.0372	β-HOMO-15→β-LUMO	0.349	L^{F}MCT
				α-HOMO-1→α-LUMO+1	0.203	MC
				β-HOMO→β-LUMO+2	0.127	MC
19	2.5990	477.05	0.0604	β-HOMO-16→β-LUMO	0.224	L^{F}MCT
				β-HOMO-15→β-LUMO	0.157	L^{F}MCT
				α-HOMO-1→α-LUMO	0.139	MC
20	2.6129	474.51	0.0361	β-HOMO-16→β-LUMO	0.428	L^{F}MCT
				β-HOMO→β-LUMO+2	0.097	MC
21	2.6676	464.78	0.0329	β-HOMO-18→β-LUMO	0.434	L^{F}MCT
				α-HOMO-2→α-LUMO	0.195	MC
				β-HOMO-1→β-LUMO+1	0.167	MC
22	2.6911	460.72	0.1313	β-HOMO-18→β-LUMO	0.426	L^{F}MCT
				α-HOMO-2→α-LUMO	0.161	MC
				β-HOMO-1→β-LUMO+1	0.149	MC
23	2.7211	455.64	0.0901	β-HOMO-1→β-LUMO+2	0.259	MC

				α-HOMO-2→α-LUMO+1	0.244	MC
				β-HOMO-17→β-LUMO	0.167	L^{F}MCT
24	2.7432	451.97	0.1225	β-HOMO-17→β-LUMO	0.447	L^{F}MCT
				α-HOMO-2→α-LUMO+1	0.162	MC
				β-HOMO-1→β-LUMO+2	0.102	MC
25	2.7730	447.11	0.0030	α-HOMO→α-LUMO+5	0.804	ML^{F}CT
				α-HOMO→α-LUMO+4	0.149	ML^{F}CT
26	2.8432	436.07	0.0016	α-HOMO-1→α-LUMO+2	0.337	MC
				β-HOMO→β-LUMO+4	0.256	MC
				α-HOMO-1→α-LUMO+3	0.18	MC
27	2.8827	430.10	0.0309	β-HOMO-20→β-LUMO	0.46	L^{F}MCT
				β-HOMO-19→β-LUMO	0.326	L^{F}MCT
28	2.9092	426.18	0.0019	β-HOMO-1→β-LUMO+4	0.251	MC
				α-HOMO-2→α-LUMO+2	0.222	MC
				α-HOMO-2→α-LUMO+3	0.111	MC
				β-HOMO-21→β-LUMO	0.098	L^{F}MCT
29	2.9234	424.11	0.0251	β-HOMO→β-LUMO+4	0.336	MC
				α-HOMO-1→α-LUMO+2	0.253	MC
				β-HOMO→β-LUMO+3	0.165	MC
30	2.9522	419.97	0.0006	β-HOMO→β-LUMO+3	0.299	MC
				α-HOMO-1→α-LUMO+3	0.154	MC
				α-HOMO→α-LUMO+6	0.142	ML^{F}CT
31	2.9630	418.44	0.0121	α-HOMO→α-LUMO+6	0.665	ML^{F}CT
32	2.9782	416.31	0.0006	α-HOMO-1→α-LUMO+4	0.357	ML^{F}CT
				β-HOMO→β-LUMO+5	0.168	ML^{F}CT
				β-HOMO→β-LUMO+6	0.161	ML^{F}CT
33	2.9989	413.43	0.0030	β-HOMO-19→β-LUMO	0.48	L^{F}MCT
				β-HOMO-20→β-LUMO	0.354	L^{F}MCT
34	3.0203	410.50	0.0029	α-HOMO→α-LUMO+7	0.823	ML^{F}CT
35	3.0374	408.19	0.0053	β-HOMO-21→β-LUMO	0.753	L^{F}MCT
				β-HOMO-1→β-LUMO+4	0.057	MC
36	3.0463	407.00	0.0150	α-HOMO→α-LUMO+9	0.467	ML^{F}CT
				α-HOMO→α-LUMO+17	0.168	ML^{F}CT
37	3.0749	403.21	0.0469	α-HOMO-1→α-LUMO+4	0.283	ML^{F}CT
				β-HOMO→β-LUMO+6	0.175	ML^{F}CT
				β-HOMO→β-LUMO+5	0.167	ML^{F}CT
38	3.0800	402.55	0.1323	α-HOMO→α-LUMO+8	0.365	ML^{F}CT
				α-HOMO-1→α-LUMO+3	0.152	MC
				β-HOMO→β-LUMO+3	0.143	MC
39	3.0912	401.09	0.1425	α-HOMO→α-LUMO+8	0.459	ML^{F}CT
				α-HOMO-1→α-LUMO+3	0.153	MC
				β-HOMO→β-LUMO+3	0.101	MC

40	3.1010	399.82	0.0337	α-HOMO-2→α-LUMO+4	0.202	ML^{F}CT
				β-HOMO-1→β-LUMO+6	0.168	ML^{F}CT
				α-HOMO→α-LUMO+9	0.125	ML^{F}CT
				β-HOMO-1→β-LUMO+5	0.109	ML^{F}CT
41	3.1139	398.16	0.0819	α-HOMO-2→α-LUMO+4	0.166	ML^{F}CT
				α-HOMO→α-LUMO+9	0.12	ML^{F}CT
				α-HOMO→α-LUMO+14	0.114	ML^{F}CT
42	3.1285	396.31	0.0156	α-HOMO→α-LUMO+10	0.625	ML^{F}CT
				α-HOMO→α-LUMO+14	0.103	ML^{F}CT
43	3.1435	394.41	0.0242	β-HOMO-22→β-LUMO	0.428	L^{F}MCT
				β-HOMO-1→β-LUMO+3	0.233	MC
				α-HOMO-2→α-LUMO+3	0.117	MC
44	3.1484	393.80	0.0019	β-HOMO-1→β-LUMO+3	0.22	MC
				β-HOMO-1→β-LUMO+4	0.124	MC
				β-HOMO-22→β-LUMO	0.086	L^{F}MCT
45	3.1540	393.10	0.0347	α-HOMO→α-LUMO+10	0.283	ML^{F}CT
				α-HOMO→α-LUMO+14	0.215	ML^{F}CT
				α-HOMO→α-LUMO+19	0.126	ML^{F}CT
46	3.1682	391.34	0.0145	α-HOMO→α-LUMO+12	0.121	ML^{F}CT
				β-HOMO→β-LUMO+6	0.113	ML^{F}CT
				α-HOMO→α-LUMO+19	0.102	ML^{F}CT
47	3.1754	390.45	0.0016	α-HOMO→α-LUMO+12	0.271	ML^{F}CT
				α-HOMO→α-LUMO+19	0.14	ML^{F}CT
				β-HOMO→β-LUMO+6	0.101	ML^{F}CT
48	3.1952	388.03	0.0663	α-HOMO→α-LUMO+16	0.345	ML^{F}CT
				α-HOMO→α-LUMO+13	0.098	ML^{F}CT
				α-HOMO→α-LUMO+18	0.093	ML^{F}CT
49	3.2003	387.41	0.0330	α-HOMO-2→α-LUMO+3	0.396	MC
				β-HOMO-1→β-LUMO+3	0.238	MC
				α-HOMO-2→α-LUMO+2	0.172	MC
50	3.2072	386.58	0.0317	α-HOMO→α-LUMO+11	0.753	ML^{F}CT
51	3.2143	385.73	0.0267	α-HOMO→α-LUMO+13	0.606	ML^{F}CT
				α-HOMO→α-LUMO+16	0.159	ML^{F}CT
52	3.2226	384.73	0.0470	α-HOMO→α-LUMO+12	0.184	ML^{F}CT
				α-HOMO→α-LUMO+14	0.171	ML^{F}CT
				α-HOMO→α-LUMO+13	0.158	ML^{F}CT
53	3.2367	383.06	0.0668	α-HOMO→α-LUMO+15	0.303	ML^{F}CT
				α-HOMO→α-LUMO+12	0.114	ML^{F}CT
				α-HOMO→α-LUMO+16	0.111	ML^{F}CT
54	3.2507	381.41	0.0094	α-HOMO→α-LUMO+15	0.137	ML^{F}CT
55	3.2590	380.44	0.0064	β-HOMO-28→β-LUMO	0.18	L^{F}MCT
				α-HOMO→α-LUMO+17	0.163	ML^{F}CT

56	3.2609	380.21	0.0064	α-HOMO-1→α-LUMO+5	0.213	ML^{F}CT
				β-HOMO-28→β-LUMO	0.167	L^{F}MCT
57	3.2689	379.28	0.1100	α-HOMO→α-LUMO+17	0.142	ML^{F}CT
				β-HOMO-28→β-LUMO	0.108	L^{F}MCT
58	3.2799	378.01	0.1163	α-HOMO→α-LUMO+18	0.194	ML^{F}CT
				α-HOMO-1→α-LUMO+5	0.133	ML^{F}CT
				α-HOMO-2→α-LUMO+4	0.118	ML^{F}CT
59	3.2834	377.61	0.0999	α-HOMO→α-LUMO+15	0.215	ML^{F}CT
				α-HOMO→α-LUMO+19	0.133	ML^{F}CT

Note: L^{F}MCT: pentafluorobenzenethiol ligand to core charge transfer, ML^{F}CT: core to pentafluorobenzenethiol ligand charge transfer, MC: core-based charge transfer.

Supplementary Table 12. Selected orbitals of Pd_1Ag_{13}.

α-HOMO-4 (-4.998 eV) 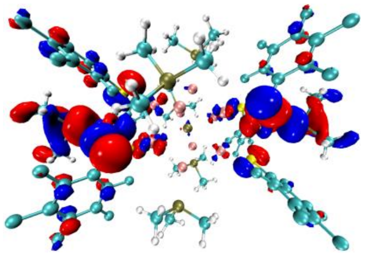	α-HOMO-3 (-4.869 eV) 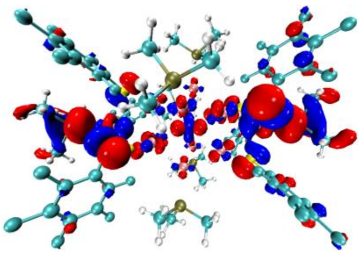	α-HOMO-2 (-4.325 eV) 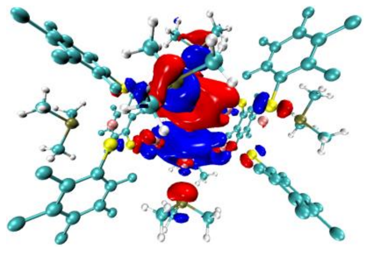
α-HOMO-1 (-4.219 eV) 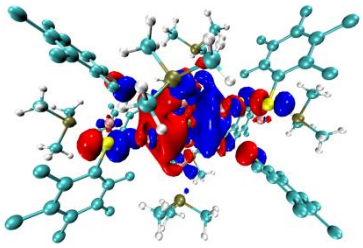	α-HOMO (-3.877 eV) 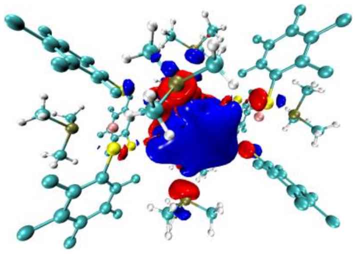	α-LUMO (-1.238 eV) 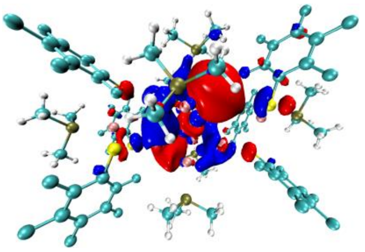
α-LUMO+1 (-1.207 eV) 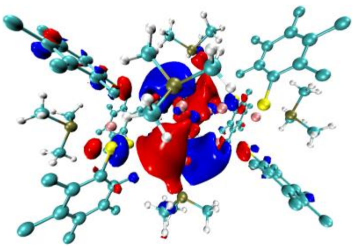	α-LUMO+2 (-0.825 eV) 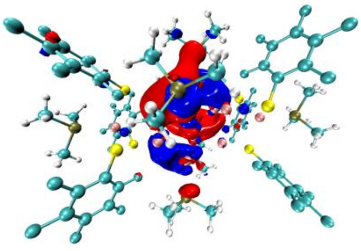	α-LUMO+3 (-0.745 eV) 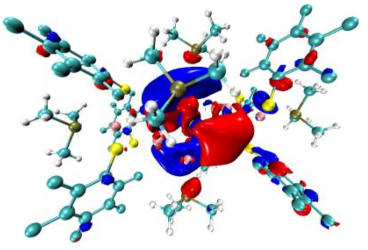

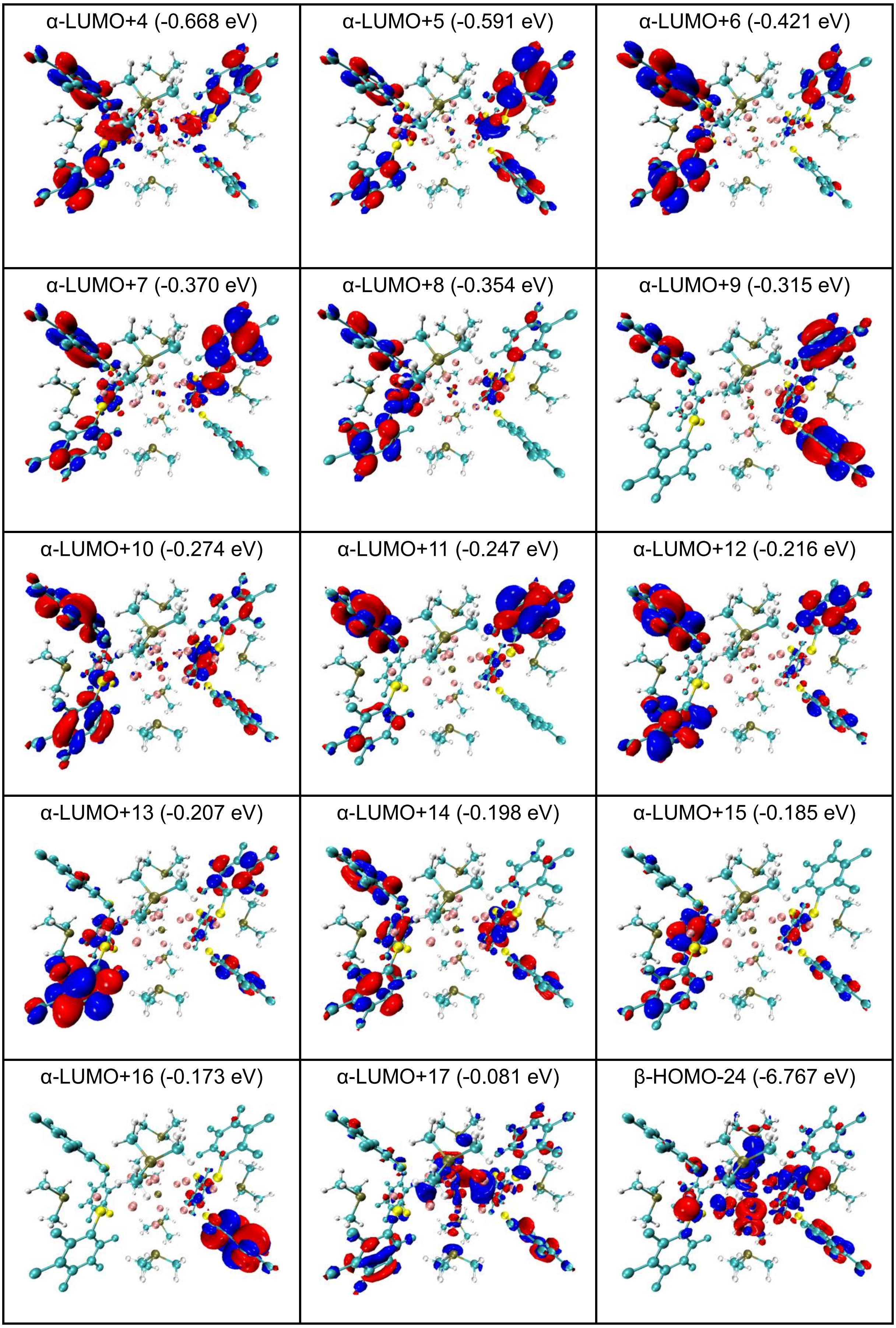
α-LUMO+4 (-0.668 eV)
α-LUMO+5 (-0.591 eV)
α-LUMO+6 (-0.421 eV)
α-LUMO+7 (-0.370 eV)
α-LUMO+8 (-0.354 eV)
α-LUMO+9 (-0.315 eV)
α-LUMO+10 (-0.274 eV)
α-LUMO+11 (-0.247 eV)
α-LUMO+12 (-0.216 eV)
α-LUMO+13 (-0.207 eV)
α-LUMO+14 (-0.198 eV)
α-LUMO+15 (-0.185 eV)
α-LUMO+16 (-0.173 eV)
α-LUMO+17 (-0.081 eV)
β-HOMO-24 (-6.767 eV)

β-HOMO-23 (-6.744 eV) 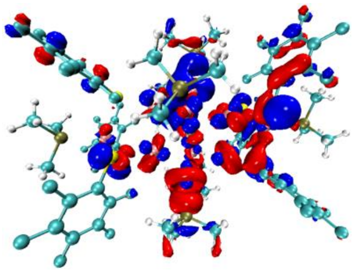	β-HOMO-22 (-6.637 eV) 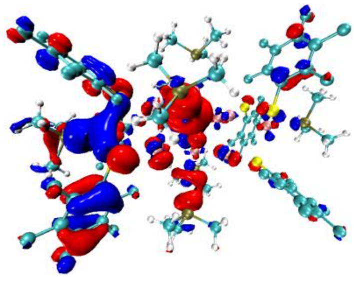	β-HOMO-21 (-6.598 eV) 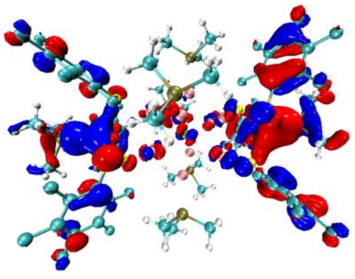
β-HOMO-20 (-6.416 eV) 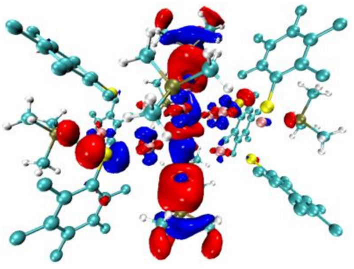	β-HOMO-19 (-6.379 eV) 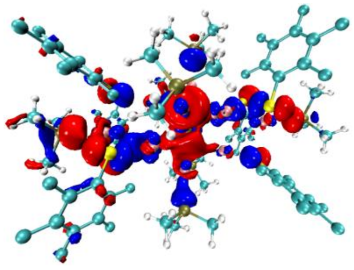	β-HOMO-18 (-6.365 eV) 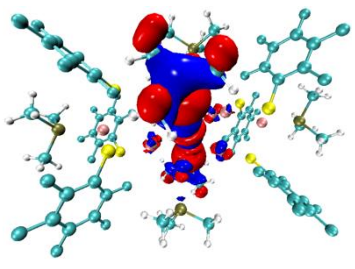
β-HOMO-17 (-6.237 eV) 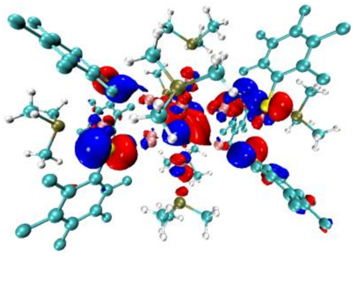	β-HOMO-16 (-6.212 eV) 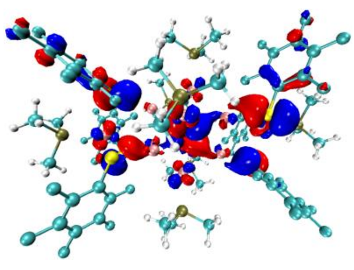	β-HOMO-15 (-6.093 eV) 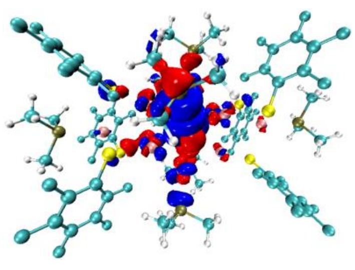
β-HOMO-14 (-5.880 eV) 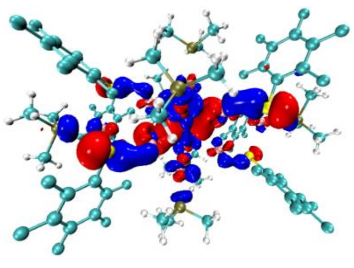	β-HOMO-13 (-5.831 eV) 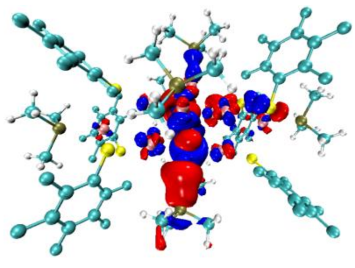	β-HOMO-12 (-5.776 eV) 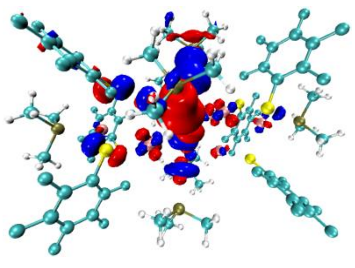
β-HOMO-11 (-5.732 eV) 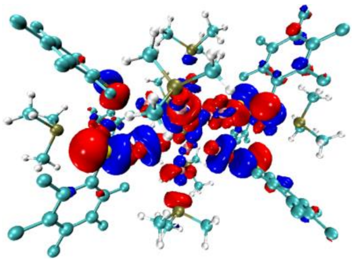	β-HOMO-10 (-5.684 eV) 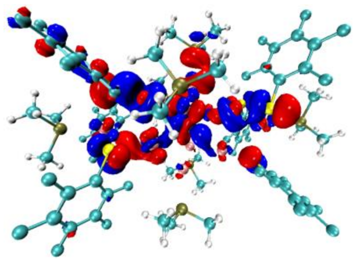	β-HOMO-9 (-5.622 eV) 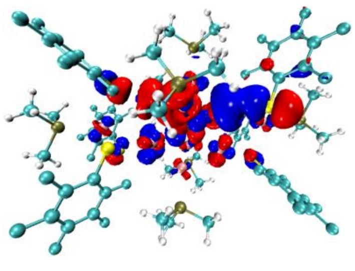

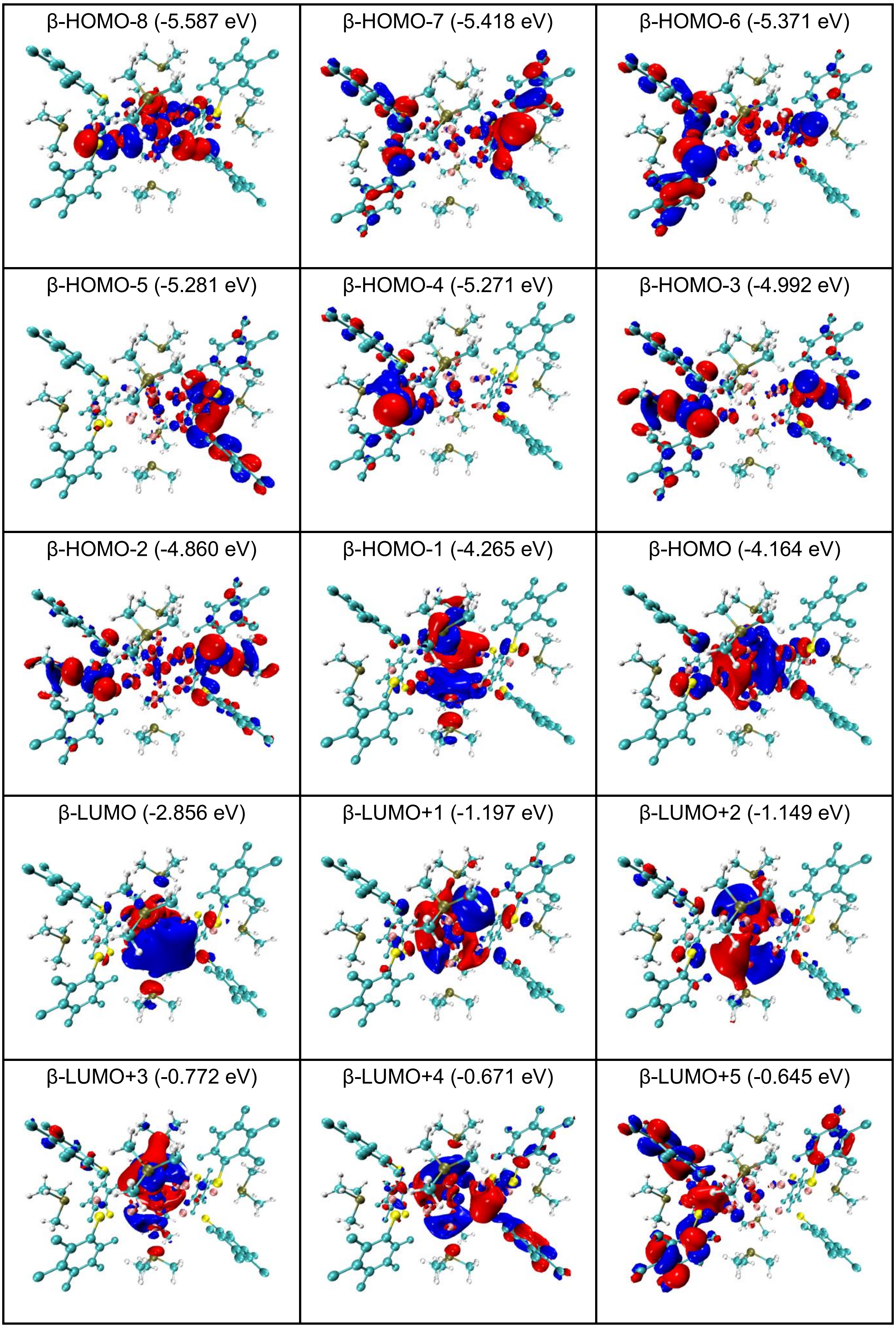
β-HOMO-8 (-5.587 eV)
β-HOMO-7 (-5.418 eV)
β-HOMO-6 (-5.371 eV)
β-HOMO-5 (-5.281 eV)
β-HOMO-4 (-5.271 eV)
β-HOMO-3 (-4.992 eV)
β-HOMO-2 (-4.860 eV)
β-HOMO-1 (-4.265 eV)
β-HOMO (-4.164 eV)
β-LUMO (-2.856 eV)
β-LUMO+1 (-1.197 eV)
β-LUMO+2 (-1.149 eV)
β-LUMO+3 (-0.772 eV)
β-LUMO+4 (-0.671 eV)
β-LUMO+5 (-0.645 eV)

β-LUMO+6 (-0.579 eV)	β-LUMO+7 (-0.415 eV)	β-LUMO+8 (-0.362 eV)
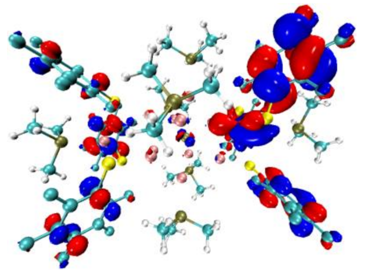	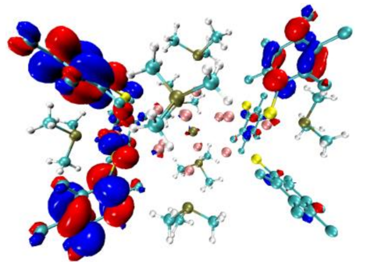	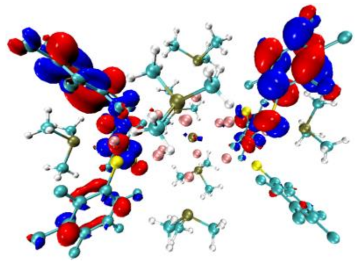

Supplementary Table 13. The selected excited states, energy, oscillator strength, and the most probable transitions of Pd_1Ag_{13} from TD-DFT calculations.

State	Energy (eV)	Energy (nm)	Oscillator strength (a.u.)	Most probable transitions	Weight of Transition	Nature of major transitions
1	1.7798	696.62	0.0002	β-HOMO-6→β-LUMO	0.251	L^FMCT
				β-HOMO-8→β-LUMO	0.238	MC
2	1.7967	690.07	0.0002	β-HOMO-8→β-LUMO	0.347	MC
				β-HOMO-4→β-LUMO	0.209	L^FMCT
				β-HOMO-9→β-LUMO	0.111	MC
3	1.8106	684.77	0.0006	β-HOMO-5→β-LUMO	0.556	L^FMCT
				β-HOMO-4→β-LUMO	0.107	L^FMCT
				β-HOMO-12→β-LUMO	0.103	MC
4	1.8479	670.95	0.0005	β-HOMO-9→β-LUMO	0.568	MC
				β-HOMO-12→β-LUMO	0.1	MC
5	1.8982	653.17	0.012	α-HOMO→α-LUMO	0.865	MC
				β-HOMO-4→β-LUMO	0.061	L^FMCT
6	1.9263	643.64	0.0024	β-HOMO-4→β-LUMO	0.346	L^FMCT
				β-HOMO-6→β-LUMO	0.266	L^FMCT
				β-HOMO-7→β-LUMO	0.093	L^FMCT
7	1.9386	639.56	0.0292	α-HOMO→α-LUMO+1	0.917	MC
8	1.9967	620.95	0.0038	β-HOMO-7→β-LUMO	0.319	L^FMCT
				β-HOMO-12→β-LUMO	0.292	MC
				β-HOMO-5→β-LUMO	0.215	L^FMCT
9	2.0238	612.63	0.0069	β-HOMO-7→β-LUMO	0.255	L^FMCT
				β-HOMO-10→β-LUMO	0.163	L^FMCT
				β-HOMO-8→β-LUMO	0.14	MC
				β-HOMO-6→β-LUMO	0.134	L^FMCT
				β-HOMO-12→β-LUMO	0.125	MC
10	2.1054	588.89	0.0023	β-HOMO-14→β-LUMO	0.238	L^FMCT
				β-HOMO-10→β-LUMO	0.198	L^FMCT
				β-HOMO-6→β-LUMO	0.136	L^FMCT

				β-HOMO-12→β-LUMO	0.095	MC
11	2.1247	583.54	0.0034	β-HOMO-13→β-LUMO	0.467	L^FMCT
				β-HOMO-7→β-LUMO	0.146	L^FMCT
				β-HOMO-6→β-LUMO	0.135	L^FMCT
12	2.1796	568.84	0.0002	β-HOMO→β-LUMO+1	0.441	MC
				α-HOMO-1→α-LUMO	0.422	MC
13	2.2059	562.06	0.0017	α-HOMO-1→α-LUMO+1	0.307	MC
				β-HOMO→β-LUMO+2	0.276	MC
				β-HOMO-10→β-LUMO	0.154	L^FMCT
14	2.2108	560.81	0.0031	β-HOMO-11→β-LUMO	0.328	L^FMCT
				β-HOMO-10→β-LUMO	0.203	L^FMCT
				α-HOMO-1→α-LUMO+1	0.127	MC
				β-HOMO→β-LUMO+2	0.094	MC
15	2.2326	555.34	0.0005	β-HOMO-11→β-LUMO	0.414	L^FMCT
				β-HOMO-14→β-LUMO	0.29	L^FMCT
				β-HOMO-9→β-LUMO	0.076	MC
16	2.2625	548.00	0.0005	β-HOMO-1→β-LUMO+1	0.438	MC
				α-HOMO-2→α-LUMO	0.376	MC
17	2.3033	538.29	0.0012	α-HOMO→α-LUMO+2	0.602	MC
				α-HOMO-1→α-LUMO	0.167	MC
				β-HOMO→β-LUMO+1	0.125	MC
18	2.3306	531.98	0.0011	α-HOMO-2→α-LUMO+1	0.509	MC
				β-HOMO-1→β-LUMO+2	0.365	MC
19	2.3405	529.73	0.0256	α-HOMO→α-LUMO+2	0.282	MC
				α-HOMO-1→α-LUMO	0.244	MC
				β-HOMO→β-LUMO+1	0.233	MC
				β-HOMO-1→β-LUMO+2	0.119	MC
20	2.3928	518.16	0.0176	α-HOMO→α-LUMO+3	0.355	MC
				β-HOMO→β-LUMO+2	0.212	MC
				α-HOMO-1→α-LUMO+1	0.184	MC
				α-HOMO-2→α-LUMO	0.13	MC
21	2.3993	516.75	0.0063	α-HOMO→α-LUMO+3	0.315	MC
				β-HOMO-15→β-LUMO	0.215	L^FMCT
				β-HOMO→β-LUMO+2	0.159	MC
				α-HOMO-1→α-LUMO+1	0.131	MC
22	2.4382	508.51	0.0725	β-HOMO-15→β-LUMO	0.33	L^FMCT
				α-HOMO-2→α-LUMO	0.117	MC
				β-HOMO-1→β-LUMO+1	0.117	MC
				β-HOMO→β-LUMO+2	0.08	MC
23	2.4966	496.61	0.0812	β-HOMO-1→β-LUMO+2	0.257	MC
				α-HOMO-2→α-LUMO+1	0.222	MC
				α-HOMO-2→α-LUMO	0.083	MC

24	2.5125	493.47	0.1422	α-HOMO→α-LUMO+3	0.236	MC
				β-HOMO-15→β-LUMO	0.173	L^{F}MCT
				β-HOMO-1→β-LUMO+1	0.148	MC
				α-HOMO-2→α-LUMO	0.128	MC
25	2.5747	481.55	0.0148	β-HOMO-16→β-LUMO	0.652	L^{F}MCT
				β-HOMO-17→β-LUMO	0.103	L^{F}MCT
26	2.5813	480.32	0.0399	β-HOMO-17→β-LUMO	0.703	L^{F}MCT
				β-HOMO-16→β-LUMO	0.092	L^{F}MCT
27	2.6273	471.91	2.0029	α-HOMO-1→α-LUMO+2	0.399	MC
				β-HOMO→β-LUMO+3	0.287	MC
28	2.6442	468.89	0.0123	α-HOMO→α-LUMO+4	0.435	ML^{F}CT
				β-HOMO-1→β-LUMO+3	0.131	MC
				α-HOMO-1→α-LUMO+2	0.1	MC
29	2.6822	462.25	0.0141	α-HOMO-2→α-LUMO+2	0.295	MC
				α-HOMO→α-LUMO+4	0.279	ML^{F}CT
				β-HOMO-1→β-LUMO+3	0.113	MC
				α-HOMO-1→α-LUMO+3	0.103	MC
30	2.7215	455.57	0.0081	β-HOMO-18→β-LUMO	0.445	L^{F}MCT
				β-HOMO→β-LUMO+3	0.164	MC
				β-HOMO-20→β-LUMO	0.1	L^{F}MCT
31	2.755	450.03	0.0752	α-HOMO-1→α-LUMO+2	0.305	MC
				β-HOMO→β-LUMO+3	0.254	MC
				β-HOMO-18→β-LUMO	0.153	L^{F}MCT
32	2.7559	449.89	0.0029	α-HOMO-1→α-LUMO+3	0.498	MC
				β-HOMO→β-LUMO+4	0.205	ML^{F}CT
				α-HOMO-2→α-LUMO+2	0.112	MC
33	2.7962	443.4	0.0206	β-HOMO-20→β-LUMO	0.38	L^{F}MCT
				β-HOMO-1→β-LUMO+3	0.144	MC
				α-HOMO-2→α-LUMO+3	0.11	MC
				β-HOMO-19→β-LUMO	0.109	L^{F}MCT
				β-HOMO-18→β-LUMO	0.099	L^{F}MCT
34	2.8180	439.97	0.0018	α-HOMO→α-LUMO+5	0.88	ML^{F}CT
35	2.8213	439.46	0.0138	β-HOMO→β-LUMO+4	0.405	ML^{F}CT
				β-HOMO-1→β-LUMO+3	0.185	MC
				α-HOMO-1→α-LUMO+3	0.165	MC
				α-HOMO-2→α-LUMO+2	0.125	MC
36	2.8409	436.43	0.0072	α-HOMO-2→α-LUMO+3	0.425	MC
				β-HOMO-1→β-LUMO+4	0.354	ML^{F}CT
				β-HOMO-20→β-LUMO	0.073	L^{F}MCT
37	2.9078	426.38	0.0166	β-HOMO-19→β-LUMO	0.476	L^{F}MCT
				β-HOMO-20→β-LUMO	0.204	L^{F}MCT
				α-HOMO-1→α-LUMO+4	0.084	ML^{F}CT

38	2.9230	424.17	0.0234	β-HOMO→β-LUMO+5	0.294	ML^{F}CT
				α-HOMO-1→α-LUMO+4	0.253	ML^{F}CT
				β-HOMO→β-LUMO+4	0.119	ML^{F}CT
39	2.9597	418.91	0.1848	α-HOMO-2→α-LUMO+2	0.197	MC
				β-HOMO-1→β-LUMO+3	0.14	MC
				β-HOMO→β-LUMO+5	0.129	ML^{F}CT
				β-HOMO-19→β-LUMO	0.113	L^{F}MCT
40	2.9969	413.71	0.0268	α-HOMO→α-LUMO+6	0.808	ML^{F}CT
41	3.0036	412.79	0.1319	β-HOMO-1→β-LUMO+4	0.369	ML^{F}CT
				α-HOMO-2→α-LUMO+3	0.29	MC
				α-HOMO-2→α-LUMO+4	0.058	ML^{F}CT
42	3.0204	410.49	0.0016	α-HOMO-3→α-LUMO	0.655	L^{F}MCT
				β-HOMO-2→β-LUMO+1	0.22	L^{F}MCT
43	3.0409	407.72	0.1557	α-HOMO-1→α-LUMO+4	0.47	ML^{F}CT
				β-HOMO→β-LUMO+5	0.341	ML^{F}CT
				α-HOMO→α-LUMO+8	0.055	ML^{F}CT
44	3.0591	405.3	0.0369	α-HOMO→α-LUMO+7	0.547	ML^{F}CT
				α-HOMO-3→α-LUMO+1	0.113	L^{F}MCT
				β-HOMO-1→β-LUMO+5	0.092	ML^{F}CT
45	3.0644	404.6	0.0084	α-HOMO-3→α-LUMO+1	0.572	L^{F}MCT
				β-HOMO-2→β-LUMO+2	0.128	L^{F}MCT
				α-HOMO→α-LUMO+7	0.084	ML^{F}CT
46	3.076	403.07	0.0133	α-HOMO→α-LUMO+8	0.404	ML^{F}CT
				β-HOMO→β-LUMO+6	0.289	ML^{F}CT
				β-HOMO→β-LUMO+5	0.054	ML^{F}CT
47	3.0789	402.69	0.0032	β-HOMO-1→β-LUMO+5	0.407	ML^{F}CT
				α-HOMO-2→α-LUMO+4	0.182	ML^{F}CT
				α-HOMO→α-LUMO+7	0.089	ML^{F}CT
48	3.0833	402.12	0.0105	β-HOMO-2→β-LUMO+1	0.656	L^{F}MCT
				α-HOMO-3→α-LUMO	0.221	L^{F}MCT
49	3.0896	401.3	0.0271	β-HOMO→β-LUMO+6	0.463	ML^{F}CT
				α-HOMO→α-LUMO+8	0.281	ML^{F}CT
50	3.1149	398.04	0.0092	α-HOMO→α-LUMO+9	0.587	ML^{F}CT
				α-HOMO→α-LUMO+7	0.056	ML^{F}CT
51	3.1253	396.71	0.0118	β-HOMO-22→β-LUMO	0.367	L^{F}MCT
				β-HOMO-23→β-LUMO	0.166	L^{F}MCT
				β-HOMO-21→β-LUMO	0.145	L^{F}MCT
				α-HOMO→α-LUMO+9	0.097	ML^{F}CT
52	3.1417	394.64	0.0288	α-HOMO-1→α-LUMO+5	0.479	ML^{F}CT
				α-HOMO-2→α-LUMO+4	0.157	ML^{F}CT
				α-HOMO→α-LUMO+9	0.098	ML^{F}CT
53	3.1428	394.5	0.0343	α-HOMO-2→α-LUMO+4	0.307	ML^{F}CT

				β-HOMO-2→β-LUMO+2	0.219	L^FMCT
				α-HOMO-1→α-LUMO+5	0.117	ML^FCT
				β-HOMO-1→β-LUMO+5	0.072	ML^FCT
54	3.1481	393.84	0.039	β-HOMO-2→β-LUMO+2	0.463	L^FMCT
				α-HOMO-1→α-LUMO+5	0.183	ML^FCT
				α-HOMO-3→α-LUMO+1	0.113	L^FMCT
				α-HOMO-2→α-LUMO+4	0.072	ML^FCT
55	3.1575	392.67	0.0226	α-HOMO→α-LUMO+10	0.65	ML^FCT
56	3.1927	388.34	0.0018	β-HOMO-1→β-LUMO+6	0.565	ML^FCT
				α-HOMO-2→α-LUMO+5	0.119	ML^FCT
				β-HOMO-1→β-LUMO+5	0.05	ML^FCT
57	3.2008	387.35	0.0035	β-HOMO-24→β-LUMO	0.323	L^FMCT
				β-HOMO-23→β-LUMO	0.294	L^FMCT
				α-HOMO→α-LUMO+11	0.106	ML^FCT
58	3.2049	386.86	0.0263	α-HOMO→α-LUMO+11	0.461	ML^FCT
				α-HOMO→α-LUMO+17	0.112	ML^FCT
				β-HOMO-23→β-LUMO	0.083	L^FMCT
59	3.2228	384.71	0.0007	α-HOMO-4→α-LUMO	0.531	L^FMCT
				β-HOMO-3→β-LUMO+1	0.208	L^FMCT
60	3.2368	383.05	0.0413	α-HOMO→α-LUMO+17	0.293	ML^FCT
				α-HOMO→α-LUMO+13	0.123	ML^FCT
				α-HOMO→α-LUMO+10	0.075	ML^FCT
				α-HOMO→α-LUMO+12	0.07	ML^FCT
61	3.2448	382.1	0.0033	α-HOMO-4→α-LUMO+1	0.393	L^FMCT
				β-HOMO-3→β-LUMO+2	0.102	L^FMCT
62	3.2523	381.22	0.0027	α-HOMO→α-LUMO+12	0.365	ML^FCT
				α-HOMO→α-LUMO+11	0.27	ML^FCT
				α-HOMO→α-LUMO+14	0.143	ML^FCT
63	3.2566	380.72	0.0003	α-HOMO→α-LUMO+14	0.612	ML^FCT
				α-HOMO→α-LUMO+12	0.106	ML^FCT
				α-HOMO→α-LUMO+13	0.10	ML^FCT
64	3.2642	379.83	0.0076	β-HOMO→β-LUMO+7	0.56	ML^FCT
				α-HOMO-1→α-LUMO+6	0.06	ML^FCT
				α-HOMO→α-LUMO+12	0.052	ML^FCT
65	3.2663	379.59	0.0083	α-HOMO-2→α-LUMO+5	0.417	ML^FCT
				β-HOMO-21→β-LUMO	0.177	L^FMCT
				β-HOMO-1→β-LUMO+6	0.15	ML^FCT
66	3.2677	379.42	0.0052	β-HOMO-21→β-LUMO	0.416	L^FMCT
				α-HOMO-2→α-LUMO+5	0.155	ML^FCT
				β-HOMO-22→β-LUMO	0.124	L^FMCT

Supplementary Table 14. The excitation energy level (in eV) and the corresponding transition molecular orbital component for the excited states of Q_1-Q_{11} in Pt_1Ag_{13}.

State	Energy (eV)	Orbital	Contribution (%)
Q_1	2.041		
Q_2	2.685	α-HOMO→α-LUMO	98.9
Q_3	2.758	β-HOMO→β-LUMO	98.7
Q_4	3.053	α-HOMO→α-LUMO+1	79.8
		β-HOMO→β-LUMO+1	14.3
Q_5	3.114	β-HOMO→β-LUMO+1	74.9
		α-HOMO→α-LUMO+1	16.7
		α-HOMO→α-LUMO+2	5.8
Q_6	3.15	α-HOMO→α-LUMO+2	79.1
		β-HOMO→β-LUMO+1	9.2
		α-HOMO→α-LUMO+3	6.4
Q_7	3.371	α-HOMO→α-LUMO+3	67.5
		α-HOMO→α-LUMO+5	6.2
		α-HOMO→α-LUMO+2	5.9
		β-HOMO-5→β-LUMO	5
Q_8	3.397	β-HOMO-5→β-LUMO	59.6
		α-HOMO→α-LUMO+3	9
		β-HOMO-7→β-LUMO	8.3
		β-HOMO-6→β-LUMO	6.3
Q_9	3.414	α-HOMO→α-LUMO+5	28
		β-HOMO-3→β-LUMO	19.9
		β-HOMO-6→β-LUMO	7.1
		β-HOMO-8→β-LUMO	6.7
		α-HOMO→α-LUMO+3	6.3
		β-HOMO-11→β-LUMO	5.6
Q_{10}	3.441	β-HOMO-1→β-LUMO	56.4
		β-HOMO-10→β-LUMO	11.9
		β-HOMO-8→β-LUMO	11.2
		α-HOMO→α-LUMO+5	5.6
Q_{11}	3.456	β-HOMO-9→β-LUMO	29.9
		α-HOMO→α-LUMO+5	20.5
		β-HOMO-4→β-LUMO	12.4
		β-HOMO-6→β-LUMO	11.7
		β-HOMO-2→β-LUMO	6.8
		β-HOMO-3→β-LUMO	5.5
