# Supplementary material for: Dual-quartet phosphorescent emission in the open-shell M1Ag13 (M = Pt, Pd) nanoclusters
Source: Nat Commun. 2024 Jul 16;15:5962. doi: 10.1038/s41467-024-50289-x (PMC11252300; doi:10.1038/s41467-024-50289-x)
Supplement: Supplementary file 3 — Description of Additional Supplementary Files [file 41467_2024_50289_MOESM3_ESM.pdf]

Description of Additional Supplementary Files:

Supplementary Data 1: The atomic coordinates of the optimized computational models of Pt_1Ag_{13} and Pd_1Ag_{13}.
